# A Novel Composite Material UiO-66-Br@MBC for Mercury Removal from Flue Gas: Preparation and Mechanism

**DOI:** 10.3390/polym16172508

**Published:** 2024-09-03

**Authors:** Zhen Zhang, Zikuo Li, Youxiang Feng, Jingxiang Yu, Xikai Zhang, Jinchao Wen, Haotian Nie, Yue Yu, Li Jia

**Affiliations:** 1 College of Electrical and Power Engineering, Taiyuan University of Technology, Taiyuan 030024, China; tyutzhangzhen@163.com (Z.Z.); 19834008532@163.com (Z.L.); 15734969860@163.com (Y.F.); jingxiangyu0819@163.com (J.Y.); zxklanyi001@163.com (X.Z.); 18835003405@163.com (J.W.); niehaotian0327@163.com (H.N.); 2 College of Economics and Management, Taiyuan University of Technology, Taiyuan 030024, China; yuyue@tyut.edu.cn

**Keywords:** MOFs, biochar, composite, Hg^0^ removal

## Abstract

To reduce the mercury content in flue gas from coal-fired power plants and to obtain high-performance, low-cost mercury adsorbents, a novel composite material was prepared by structural design through the in situ growth method. Functionalization treatments such as the modification of functional groups and multilayer loading of polymetallic were conducted. These materials include the MOF material UiO-66 and modified biochar doped with Fe/Ce polymetallic, both of which contain unsaturated metal centrals and oxygen-containing functional groups. On the basis of obtaining the effects of adsorption temperature and composite ratio on the Hg^0^ removal characteristics, coupling and synergistic mechanisms between the various types of active centers included were investigated by using a variety of characterization and analysis tools. The active adsorption sites and oxidation sites were identified during this process, and the constitutive relationship between the physicochemical properties and the performance of Hg^0^ removal was established. The temperature-programmed desorption technique, Grand Canonical Monte Carlo simulation, and adsorption kinetic model were employed to reveal the mechanism of Hg^0^ removal. The results showed that the UiO-66-Br@MBC composite adsorbent possessed an excellent Hg^0^ removal performance at adsorption temperatures ranging from 50 to 250 °C, and targeted construction of adsorption and oxidation sites while maintaining thermal stability. The Hg^0^ removal by the composites is the result of both adsorption and oxidation. The micropores and small pore mesopores in the samples provide physical adsorption sites. The modified biochar acts as a carrier to facilitate the full exposure of the central metal zirconium ions, the formation of more active sites, and the process of electron transfer. The doping modification of the Br element can enhance the overall redox ability of the sample, and the introduced Fe and Ce polymetallic ions can work in concert to promote the oxidation process of Hg^0^. The excellent regulation of the ratio between adsorption and oxidation sites on the surface of the composite material finally led to a significant boost in the samples’ capacity to remove Hg^0^.

## 1. Introduction

Anthropogenic activities account for about 40% of mercury pollution in the atmosphere. China, as one of the first parties to the Minamata Convention on Mercury, has been facing the urgent task of mercury emission reduction. Mercury emissions from coal combustion in China account for 50% of the total mercury emissions, and coal burned in the electricity sector represents 60% of the total coal production. Coal-fired power plants are thought to be the primary source of mercury emission pollution at this time [1]. Flue gas contains three main forms of mercury: elemental mercury(Hg^0^), oxidized mercury(Hg^2+^), and mercury in particulate form(Hg^P^). Of them, Hg^P^ and Hg^2+^ can be synergistically removed by existing pollution-control devices such as WFGDs in power plants; however, Hg^0^ can be removed only via an activated carbon injection, in combination with specialized equipment, since it is poorly soluble in water [2]. Therefore, exploring and researching the efficient and green way of mercury removal has become a work of considerable practical application value and a major global demand in the fields of energy and environment.

Currently, metal–organic frameworks(MOFs) have great potential for flue gas-pollutant removal; MOFs are coordination porous materials formed by linking inorganic metal elements(including metal ions or metal clusters) as nodes with organic ligands through self-assembly. Because of their plentiful unsaturated metal sites, uniform and adjustable pore structure, and potential for functionalized modification of chemical and physical properties through targeted structural design, MOFs have garnered a great deal of interest from researchers. At this point, UiO-66 is considered a typical representative of MOF materials due to its high physicochemical stability and ease of functionalization modification. It has a variety of applications in the fields of pollutant adsorption and gas separation and storage [3]. Moreover, the unsaturated zirconium-based active metal sites exposed on the surface of UiO-66 contribute to the adsorption of gaseous Hg^0^ and thus have the potential for mercury removal.

Previous research has demonstrated that functionalized MOF materials formed by grafting halogen functional groups to UiO-66 can significantly enhance the removal efficiency of elemental mercury from the flue gas of coal-fired power plants. Zhao et al. [4] found that the incorporation of-Br-based functional groups can play a facilitating role in the removal of Hg^0^ compared to the introduction of-NO_2_ and-NH_2_. However, the use of traditional single MOF materials as mercury removal agents is still facing many problems. These issues include the materials’ poor physical and chemical stability, their ability to readily agglomerate metal-based nanoparticles, and the fact that they have only one active site available for reaction, which in turn limits their application scenarios for gaseous mercury pollution [5]. To address the aforementioned issues, the creation of MOF-based composites has sparked a great deal of interest in research among academics. By compositing MOFs with components such as carbon nanotubes, graphene oxide, and activated carbon, MOF-based composites not only address the shortcomings of the current MOFs used alone but also introduce new and expanded functions [6].

Currently, biochar, which is the same carbon-based material as activated carbon, is produced by the pyrolysis of biomass and has a complex pore structure and good surface chemical properties. The research on the use of biomass for the removal of flue gas pollutant elemental mercury has been widely carried out. Marta Marczak-Grzesik et al. [7] considered the use of corn straw char(CS-400) and brominated corn straw char(CS-400-Br) as a low-cost organic adsorbent as an effective alternative to expensive dust-activated carbon. The addition of bromine to raw biochar(CS-400) resulted in a material with a higher adsorption potential, accompanied by a greater amount of Hg^0^ to be oxidized. Gong Ruhao et al. [8] synthesized a Mn/Cu co-doped mesoporous carbon adsorbent(MnCux-C) using a hard template method, and the experimental results showed that Mn doping in mesoporous carbon is a favorable means of improving mercury removal performance and was able to increase the Hg^0^ capture rate from 6% to 89%. Jia et al. [9] prepared biochar from agricultural waste(walnut shells) and modified it with multi-metal layer loading, resulting in an enhanced Hg^0^ removal performance. The synergistic effects among the doped metals significantly improved the sample’s performance, thereby compensating for the drawbacks of biomass with low calorific value and inefficient standalone utilization. Consequently, it is possible to create MOF-based composites using UiO-66 materials that have been specifically modified by-Br functional groups to target the fundamental characteristics of biomass. This improves the efficiency of mercury removal while drastically lowering the preparation costs of MOF-based adsorbents. Simultaneously, this type of biomass serves as a valuable raw material for the production of an affordable mercury removal agent, contributing to the “waste to detoxification” process and expanding the potential applications of MOFs in line with the country’s low-carbon and green development strategy.

In summary, the difficulties and pain points faced by traditional MOF materials as mercury adsorbents, such as their poor physical and chemical stability, single active site, high preparation cost, etc., make it difficult to achieve the efficient removal of Hg^0^. In this paper, based on the modified biochar doped with Fe/Ce bimetallic and MOF material UiO-66, both of which contain unsaturated metal centers and oxygen-containing functional groups, the composite adsorbents of UiO-66-Br and Fe/Ce modified biochar were prepared by structural design through the in situ growth method based on the formation of a new pore structure and surface chemical properties. On the basis of obtaining the effects of adsorption temperature and composite ratio on the Hg^0^ removal characteristics, the microscopic properties of the composites before and after adsorption were investigated by using a variety of characterization and analysis tools. The active adsorption sites and oxidation sites were identified during this process, and the constitutive relationship between the physicochemical properties and the performance of Hg^0^ removal was established. The temperature-programmed desorption technique, Grand Canonical Monte Carlo simulation, and adsorption kinetic model were adopted to reveal the Hg^0^ removal mechanism, seeking to create new opportunities for the use of MOFs and biomass to develop a more all-encompassing approach to the treatment of mercury pollution.

## 2. Materials and Methods

### 2.1. Preparation of Samples

#### 2.1.1. UiO-66/UiO-66-Br

Firstly, 3 mmol of zirconium tetrachloride (ZrCl_4_, 99%) was dissolved in 150 mL of N,N dimethylformamide (DMF, 99%) solution and stirred by a magnetic stirrer for 30 min; then, 3 mmol of terephthalic acid (C_8_H_6_O_4_, 99%) was added. After the solution was clarified, the UiO-66 precursor solution was obtained and transferred to a 250 mL hydrothermal synthesis reactor with PTFE lining, heated at 120 °C for 24 h, and then naturally cooled to room temperature; finally, the sample was washed three times with DMF and anhydrous ethanol (EtOH, 99%), and then the sample obtained was dried in an oven at 120 °C for 24 h, and then removed and ground to obtain a white powdery UiO-66 sample. Similarly, the ligand terephthalic acid was replaced with 2,5-dibromoterephthalic acid (C_8_H_4_Br_2_O_4_, 99.46%), and a white powdery UiO-66-Br sample could be obtained according to the above procedure.

#### 2.1.2. Modified Biochar

Previous studies have found that walnut shells, as an agricultural waste, have more excellent mercury removal properties than other biomasses, and the production of walnuts in China ranks first in the world. Walnut shells with particle sizes between 58 and 75 μm were selected as the raw material. Using the co-precipitation method and the high temperature conditions generated by the fixed bed, the multi-metal loading and biomass pyrolysis coke-making processes were integrated to achieve multi-metal functionalization modification of biochar. This resulted in modified walnut shell biochar, with loading mass fractions of Fe/Ce of 10% and 4%, respectively, and samples labeled as MBC. For detailed preparation procedures, please refer to the Supplementary Material.

#### 2.1.3. UiO-66-Br@MBC Composites

The UiO-66-Br@MBC composites were ultimately produced by following the steps for the preparation of UiO-66-Br. This was accomplished by using the in situ growth method, which involved adding the MBC samples and magnetically stirring them for one hour to form the composite precursor solution after obtaining the UiO-66-Br precursor solution. The prepared composites were labeled as UiO-66-Br@MBC according to the mass ratio of UiO-66-Br to MBC(MOFs/MBC = 9:1, 5:1, 2:1, 1:1, 1:2, 1:5, and 1:9).

### 2.2. Characterization

The crystalline phase structure of the samples was characterized by a BRUKER D8 ADVANCE series X-ray diffractometer (BRUKER, Karlsruhe, Germany) with a scanning speed of 10 °/min. The micro-morphology of the samples was investigated by a Hitachi SU8010 electron microscope (Hitachi, Tokyo, Japan) operated at 5 kV. The weight loss of the samples was investigated by a STA 6000 synchronous thermal analyzer (NETZSCH, Selb, Germany) with a temperature range of room temperature up to 800 °C under N_2_ atmosphere, with a temperature increase rate of 10 °C/min. The pore structure of the samples was analyzed by a Micromeritics ASAP2460 Specific Surface and Porosity Analyzer (Micromeritics, Norcross, GA, USA). The temperature range was from room temperature to 800 °C, and the temperature increase rate was set at 10 °C/min under N_2_ atmosphere; the chemical functional groups on the surface of the samples were characterized by using Nicolet iS20 FTIR (Thermo Fisher, Waltham, MA, USA) with the number of scans being 32 and the scanning range being from 400 to 4000 cm^−1^. A K-Alpha X-ray photoelectron spectrometer (Thermo Fisher, Waltham, MA, USA) was used to analyze the elemental valence states on the surface of samples.

### 2.3. Experiment System

**Figure 1:**
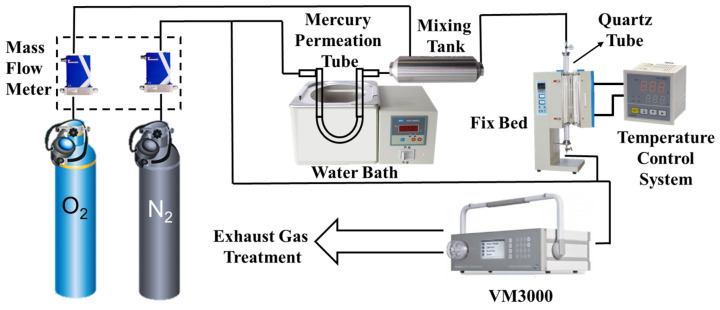
Fixed-bed mercury adsorption experiment system.

The fixed-bed Hg^0^ removal system mainly consists of a Hg^0^ generator, a gas distribution system, a fixed-bed reactor, a VM3000 mercury continuous on-line monitor produced by the MI company (North Rhine, Westphalia, Germany), and a tail gas absorption device. As shown in Figure 1, the VM3000 mercury continuous on-line monitor was used to measure the mercury concentration at the outlet of the fixed-bed reactor, with a sampling interval of 1 s and a sample loading of 0.1 g. With the flue gas after Hg^0^ removal from the sample, the corresponding mercury removal characteristics could be obtained by passing the flue gas into the VM3000. The mercury vapor was generated by a mercury permeation tube placed inside a U-shaped borosilicate glass tube, and the temperature of the water bath was adjusted to ensure that the inlet Hg^0^ concentration was maintained at a stable level of 90 μg/m^3^ during the experimental process. According to the inlet gas requirement of the VM3000 instrument, the total flow rate of the experimental gases was set to 1.4 L/min, which consisted of the carrier gas and the equilibrium gas, with the flow rate of N_2_ carrying the Hg^0^ of 430 mL/min, the flow rate of O_2_ of 70 mL/min, and the flow rate of equilibrium gas(N_2_) at the inlet of the fixed bed of 900 mL/min.

Before the Hg^0^ removal experiment, the experimental gas was bypassed into the VM3000 for monitoring, and after the Hg^0^ concentration was stabilized for 30 min, it was switched and connected to the fixed-bed reactor for on-line monitoring of the outlet mercury concentration. To prevent Hg^0^ from condensing on the wall of the pipeline due to the low temperature, all pipelines and tee parts were made of Teflon material. The tail gas during the experiment was treated using KI-modified coconut shell-activated carbon.

The cumulative amount of Hg^0^ removed per unit mass of sample, *q*, and removal efficiency, *η*, were used as the evaluation index during the Hg^0^ removal performance experiments, and the equations are as follows:

(1) $$q=\frac{F}{m}\sum_{0}^{t}\left(C_{\mathrm{in}}-C_{\mathrm{out}}\right)$$

(2) $$\eta =\frac{C_{\mathrm{out}}}{C_{\mathrm{in}}}\times 100\%$$

where *q* is the cumulative amount of Hg^0^ removed, ng/g; *F* is the flow rate, L/s; *m* is the sample mass, g; *t* is time, s; and *C*_in_ and *C*_out_ are the inlet Hg^0^ concentration and outlet Hg^0^ concentration, ng/L, respectively.

In the temperature-programmed desorption experiment, the sample after the mercury removal experiment was placed back into the fixed-bed adsorption system, and the total gas flow rate was maintained at 1.4 L/min. Under the condition of N_2_ atmosphere, the heating rate is 10 °C/min. The fugitive morphology of the removed mercury on the surface of the sample was obtained by monitoring the outlet mercury.

### 2.4. GCMC Simulation

Based on the results of experiments on the mercury adsorption properties of samples, the adsorption behavior of Hg^0^ in UiO-66, as well as UiO-66-Br microporous spaces, was studied, and a 2 × 2 × 2 supercell of MOF crystal was constructed. The crystal structure of the UiO-66 material was constructed based on the corresponding standard cell model from the Cambridge Crystallographic Data Centre (CCDC); the crystal structure was modified by doping Br elements to obtain the UiO-66-Br crystal configuration. During the study of the adsorption–diffusion mechanism of Hg^0^ using GCMC simulation, aiming at investigation of the gas–solid adsorption reaction process of gaseous Hg^0^, the UFF was selected for this simulation. The environmental temperature and the pressure were 373 K and 100.0 kPa, respectively. The ratios of mercury atom exchange, conformation isomerization, rotation, translation, and regeneration were set at 2, 1, 1, 1, and 0.1, respectively. The electrostatic interaction between the mercury atoms and UiO-66 was described by the Ewald summation method with an accuracy of 10^−4^ kcal/mol, and the van der Waals interaction was calculated using the atom-based method with a cutoff distance of 15.5 Å. The first 1 × 10^5^ moves were used for equilibration, and the subsequent 1 × 10^6^ moves were used for production. All the simulations were performed using the Forcite and Sorption modules of the Material Studio 2019.

## 3. Results

### 3.1. Physical and Chemical Characteristics

#### 3.1.1. Crystal Structure

**Figure 2:**
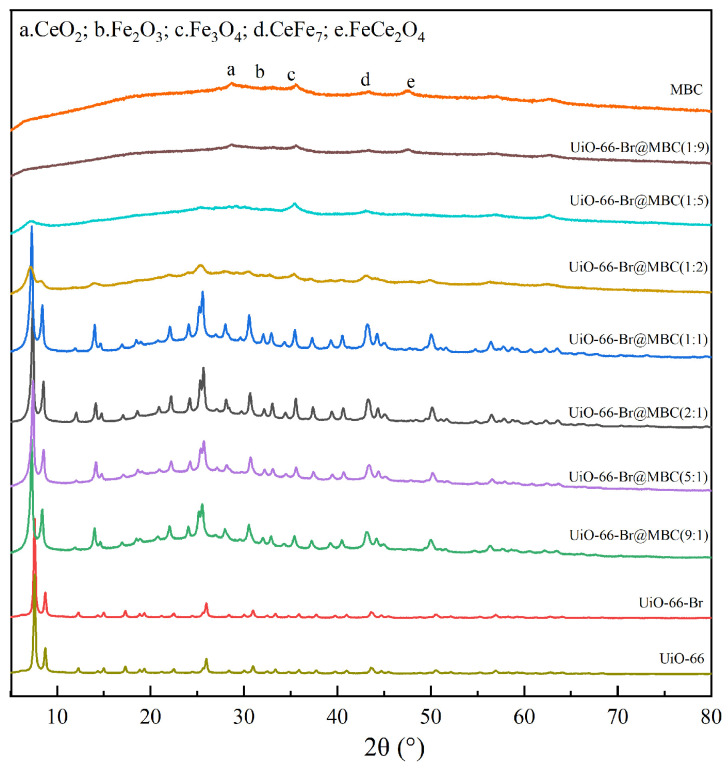
Crystalline phase structure of samples.

The material composition and crystalline phase structure of the samples were investigated to determine the constitutive relationship between the physicochemical characteristics and mercury removal performance of the UiO-66-Br-based modified biochar composite adsorbent. The findings are displayed in Figure 2. The UiO-66 samples showed obvious diffraction peaks at 7.4°, 8.6°, and 25.6°, which corresponded to the (111), (002), and (224) crystalline planes, respectively [10], indicating that the synthesized samples have obvious crystal structure properties of MOF materials and verifying the correctness of the preparation process. The framework structure of UiO-66 remains unaltered by the doping alteration of Br, allowing it to retain its topology. For biochar and its modified samples of amorphous substances, there are a certain number of graphite microcrystalline structures before modification, without other obvious diffraction peaks, and only a continuous broad peak of amorphous carbon appeared near 25° [11]. After polymetallic doping, the samples’ degree of graphitization decreases. Physical phase analysis reveals that a variety of metal oxides are endowed on their surface. The presence of these substances can complement the adsorption sites on the surface of the biochar and improve its chemisorption capacity. The UiO-66-Br@MBC(MOF/MBC = 9:1, 5:1, 2:1, and 1:1) composites have obvious crystalline structures. The matching diffraction peaks’ positions are nearly identical to those of the UiO-66-Br samples, and the MBC’s graphite microcrystalline structure is also preserved. Zr ions with smaller radii can more readily enter CeO_2_ crystals during the composite process, producing a Zr-Ce solid solution. This results in the formation of more lattice defects and a high dispersion of metal oxides, like Fe_2_O_3_ and Fe_3_O_4_, on the sample surface which work in collaboration to facilitate the removal of Hg^0^. Furthermore, it is evident that the composite adsorbent undergoes a crystallization passivation effect when the composite ratio of MBC is higher than 50%. This phenomenon slows down grain growth, which is associated with a decrease in crystal size, and results in the appearance of distinctive diffraction peaks that are linked to FeCe_2_O_4_ with a spinel structure. This is because, during the composite preparation process, Ce^4+^ undergoes an orbital hybridization cross-linking reaction with Fe^3+^ and forms a double-oxygen coordination, which has a stronger activation effect than that of the single-oxygen coordination of Zr^4+^. At the same time, the sample contains a large number of variable-valency transition metal ions; thus, a large number of cation vacancies and unsaturated reactive sites conducive to the adsorption of Hg^0^ are constructed on the surface of the samples. Moreover, this AB_2_O_4_-type spinel composite oxide can also undergo a one-electron reduction to form O-radicals, which can significantly enhance the Hg^0^ removal performance of the sample. Therefore, an excellent conformational relationship was established between the crystalline phase structure of UiO-66-Br@MBC and the Hg^0^ removal performance. However, the diffraction peak intensities and crystallinity degree gradually weakened as the composite proportion of MBC increased, indicating that the excessive introduction of MBC affects the evolution and molding of UiO-66-Br. The composites were also unable to form an obvious crystalline phase structure. The agglomerate-type reactive sites formed were not conducive to Hg^0^ removal.

#### 3.1.2. Surface Chemical Characteristics

**Figure 3:**
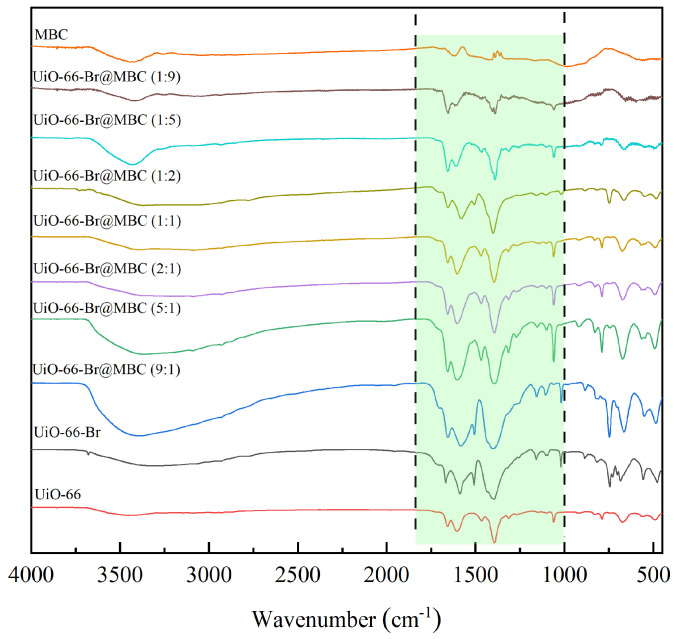
Surface chemical properties of samples.

The FTIR characterization results of the MBC, MOFs, and UiO-66-Br@MBC composites synthesized through the in situ growth method are shown in Figure 3. The green shade part represents the presence of oxygen-containing functional groups. For UiO-66, the broad peak at around 3400 cm^−1^ corresponds to the stretching vibration of the O-H bond; the peak at 1655 cm^−1^ is attributed to the C=O group in the organic ligand terephthalic acid in the MOFs material; the peaks at 1600 cm^−1^ and 1384 cm^−1^ were attributed to the O-C-O asymmetric and symmetric stretching features of terephthalic acid in the ligand, respectively, while the peak at 1062 cm^−1^ was the C-H deformation mode; in addition, the peaks caused by-OH and-CH vibrations in the benzene ring of the ligand clearly appeared at 785 cm^−1^ and 734 cm^−1^, and stretching vibration peaks attributed to Zr-O appeared at 553 cm^−1^ and 478 cm^−1^ [12]. All of the above results can verify that the UiO-66 material was synthesized efficiently. In the infrared spectrum of UiO-66-Br, C-Br functional groups at 1042 cm^−1^ and 672 cm^−1^ can be clearly found, indicating that Br can be successfully grafted into the electron-rich unsaturated carbene ring. The structure of UiO-66, through the occurrence of aromatic electrophilic substitution reactions and the formation of positively charged π-complexes, is conducive to the Hg^0^ removal. For the composite material, the overall peak intensity was higher in the vibrational region of oxygen-containing functional groups in the wavelength band of 1800 cm^−1^~1000 cm^−1^. During the composite preparation of MBC and UiO-66-Br, the latter’s abundant oxygen-containing functional groups can be combined with the unstable Zr^4+^ ions in UiO-66-Br. Additionally, the doped Fe/Ce metal ions can form metal–ligand hydroxyl functional groups (M-OH) with the functional groups in the organic ligands of UiO-66-Br through coordination and hydrogen bonds. It enhances the dispersion of metal ions by giving Hg^0^ additional chemically active sites, which not only help to strengthen the anchoring bond between UiO-66-Br and MBC but also act as an intermediary in the Hg^0^ oxidation reaction that follows. In addition, since the composites can maximally retain the original graphite microcrystalline structure of the MBC, the lattice oxygen in the metal oxides (ZrO_2_, Fe_2_O_3_, Fe_3_O_4_, CeO_2_, etc.) will re-oxidize the edges of the graphite microcrystalline structure and then generate more oxygen-containing functional groups, which act as the chemical adsorption sites for Hg^0^ to enhance the adsorption capacity of the composites. Hg^0^ was assigned to the surface of the sample as organic mercury(Hg-OM) by undergoing a complexation reaction.

#### 3.1.3. Pore Structure

To further investigate the effect of the composite preparation on the adsorption sites of UiO-66-Br-based modified biochar, the pore structure of the samples was investigated, and the obtained pore structure parameters are shown in Table 1. Among them, the UiO-66 sample has the largest BET specific surface area (1083.52 m^2^/g), has a high pore abundance, and is mainly dominated by micropores and mesopores. As a MOF material, the well-developed pore structure not only provides abundant reaction sites but also facilitates good dispersion of sites with a larger reaction area. A higher pore volume also improves the diffusion rate and reduces the mass transfer resistance of Hg^0^ in the sample. The effective contact between Hg^0^ and the active site is promoted, which in turn facilitates the gas–solid reaction. In contrast, the specific surface area, pore volume, and average pore diameter of the UiO-66-Br sample decreased, and this decrease was attributed to the fact that the original three-dimensional pore–cage spatial structure of UiO-66 would be clogged during the modification process of introducing functional groups, leading to a decrease in the physical adsorption performance, whereas the MBC had the largest average pore diameter and the highest percentage of macropores as a means of providing a diffusion channel for Hg^0^ to enter the internal pore. Although it is beneficial to reduce the along-stream resistance of flow diffusion and enhance the mass transfer diffusion rate, the proportion of micropores and smaller-pore-size mesopores as the active physical adsorption sites at the early stage of the reaction is low, so the Hg^0^ removal efficiency is not optimal. The specific surface area and pore volume exhibit an increasing and then decreasing trend as the MBC composite ratio increases. This is because more UiO-66-Br will grow in the MBC pore channels and form more mesopores during the composite process, which will optimize the pore structure. With the increasing content of various metal ions and metal oxides introduced by MBC, the process of its own oxidizability enhancement is correlated with the evolutionary process of the development of the pore structure of the biochar, which plays the role of a carrier [13]. Among them, when the composite ratio of MBC did not exceed 50%, the boundary layer of the internal pores of the sample would undergo oxidative ablation, which would increase the specific surface area and pore volume of the sample. With the largest percentage of mesopores and micropores, the UiO-66-Br@MBC(1:1) sample offered a large number of Hg^0^ physical adsorption sites early in the reaction and matched the highest initial adsorption rate, both of which would be advantageous to the ensuing oxidation process and result in highly efficient adsorption. However, with increasing the composite ratio, the pore structure parameters of the samples began to decline, and from the previous section, it is known that the excess MBC affects the crystal molding process of UiO-66-Br. The N_2_ adsorption–desorption isotherms and BJH pore-size distribution of samples are provided in Section S8 of the Supplementary Materials.

#### 3.1.4. Microscopic Morphology

**Figure 4:**
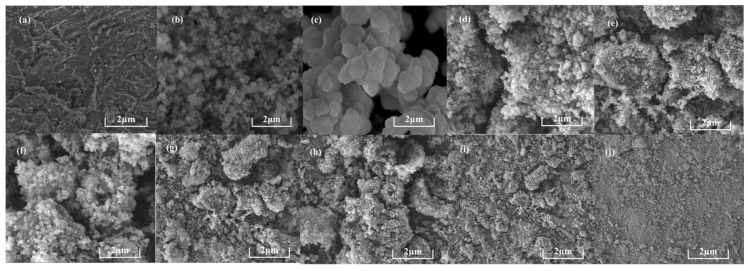
Microscopic morphology of samples: (**a**) MBC; (**b**) UiO-66; (**c**) UiO-66-Br; (**d**) UiO-66-Br@MBC(9:1); (**e**) UiO-66-Br@MBC(5:1); (**f**) UiO-66-Br@MBC(2:1); (**g**) UiO-66-Br@MBC(1:1); (**h**) UiO-66-Br@MBC(1:2); (**i**) UiO-66-Br@MBC(1:5); and (**j**) UiO-66-Br@MBC(1:9).

The SEM characterization results of MBC, MOFs, and the UiO-66-Br@MBC composites obtained through the in situ growth method are shown in Figure 4. It was found that the surface of MBC did not form a large number of endowments with a regular-shaped structure, and there was a more developed pore structure. Due to the loading of polymetallic ions, a certain degree of agglomeration occurred on the surface, and the overall flaky projection was presented. UiO-66 and UiO-66-Br, as the materials of MOFs, both showed an anisotropic nucleation of a homogeneous hexahedral shape with a high degree of crystallization. UiO-66-Br can keep its original crystal structure characteristics in composites with relatively low MBC proportions. In addition, UiO-66-Br crystals grow on the surface of the modified biochar framework and enter the MBC pore structure. UiO-66-Br was unable to maintain its original crystal structure and became dispersed in the carriers in a chaotic and disordered manner. This occurred particularly when the proportion of composites exceeded 50%. As the proportion of MBC composite continued to increase, the cubic grains on the sample surface gradually changed to spherical particles, and the size of the grains gradually shrank. The above results are in agreement with the microscopic characterization obtained in the previous section.

#### 3.1.5. Thermal Stability

**Figure 5:**
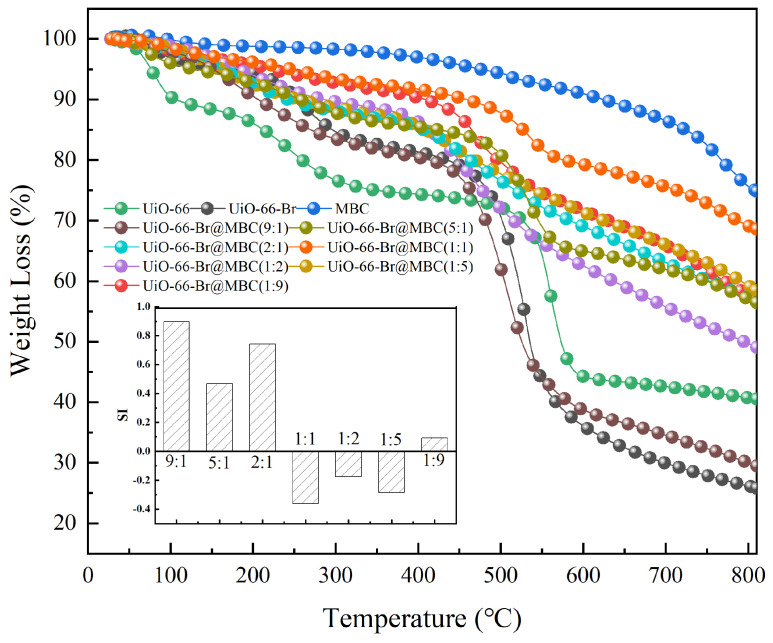
Weight-loss properties of samples.

Due to the relatively weak bonding between the central metal and the organic ligand of the MOF material, it is difficult to maintain the activity for a long time during the high-temperature heterogeneous catalytic oxidation reaction. This structural instability severely limits the removal of heavy-metal mercury pollutants. Therefore, the thermal stability characteristics of the samples obtained under different preparation conditions are investigated in this section to explore their potential for practical industrial applications, and the results are shown in Figure 5. For the UiO-66 and UiO-66-Br samples, as MOFs, the pyrolysis curves obtained are basically similar, and both contain five weight-loss stages with distinctly different characteristics. Among them, the weight loss of the UiO-66 below 120 °C mainly corresponds to the precipitation process of the contained free water molecules and anhydrous ethanol, with a mass loss of about 12%; the mass loss of the sample between 120 and 350 °C is about 13%, which is due to the volatilization and release of the unreacted organic solvent DMF residual in MOFs. The mass loss of the sample in the range of reaction temperature between 350 and 500 °C shows a platform layer. This stage results in almost no weight loss, indicating that UiO-66 can maintain good thermal stability in this temperature region. The main weight-loss stage occurs when the temperature continues to increase up to 600 °C and the supporting framework completely collapses due to the decomposition of organic ligands, corresponding to a mass loss of about 30%. With the further increase in temperature, the sample almost stops losing weight, and a stable derived carbon material forms along with the metal oxide ZrO_2_ scattered on the sample surface [14]. Similarly, below 450 °C, UiO-66-Br loses roughly 20% of its mass, and the structure starts to collapse with a sharp drop in weight. It can be seen that the modification of the Br has less of an effect on the original thermal stabilization of the UiO-66.

MBC was obtained under the high temperature condition of 600 °C; it was thermally stable with low weight loss. As for the UiO-66-Br-based modified biochar adsorbents with different composite ratios, the weight-loss curves were all intermediate between those of UiO-66-Br and MBC, and all of them were able to maintain good thermal stability up to 450 °C. In particular, the mass loss of the UiO-66-Br@MBC(1:1) was the lowest and slowest weight-loss rate of all the composite materials due to the strong interaction between the MBC and MOF materials during their composite process. To further quantitatively investigate the intensity of the interaction between UiO-66-Br and MBC and the corresponding mechanisms, the theoretical values of TG for different composite ratios were calculated by Equation (3), and the *SI* values of the interaction index obtained through Equation (4) were used to reveal the mechanism of facilitating or inhibiting effects in the process of pyrolysis evolution. The distribution of deviations from the theoretical and experimental TG curves was investigated by means of the interaction diagrams of the composites, and it can be found that the *SI* values of the UiO-66-Br@MBC(MOF/MBC = 1:1, 1:2, and 1:5) composites are all less than 0. In particular, the lowest *SI* value of-0.286 was obtained for UiO-66-Br@MBC(1:1), indicating that the overall pyrolysis process of the samples was strongly inhibited, and, thus, their thermal stability was greatly improved.

(3) $$TG_{\mathrm{cal}}^{i}=x_{\mathrm{MOF}}*TG_{\mathrm{MOF}}^{i}+x_{\mathrm{MBC}}*TG_{\mathrm{MBC}}^{i}$$

(4) $$SI=(\sum_{i=1}^{n}\left(TG_{\exp}^{i}-TG_{\mathrm{cal}}^{i}\right))/(nTG_{\mathrm{cal}}^{\mathrm{mean}})$$

where $TG_{\mathrm{MOF}}^{i}$ is the experimental TG value of UiO-66-Br sample, %; $TG_{\mathrm{MBC}}^{i}$ is the experimental TG value of MBC, %; $TG_{\mathrm{cal}}^{i}$ and $TG_{exp}^{i}$ are the theoretical and experimental TG values of composites under different ratios, %; $TG_{\mathrm{cal}}^{\mathrm{mean}}$ represents the average values of the theoretical TG values of composites under different ratio conditions, %; *x*_MOF_ and *x*_MBC_ are the mass ratios of UiO-66-Br and MBC in the composite process, %, respectively; and i and *n* represent the location and number of sampling points, respectively.

### 3.2. Hg^0^ Removal Performance

#### 3.2.1. Effect of Adsorption Temperature

**Figure 6:**
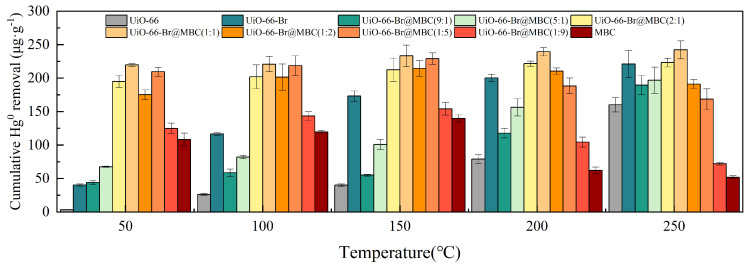
Effect of adsorption temperature on Hg^0^ removal performance.

The trend of mercury removal performance of MOFs and their composites with increasing adsorption temperature is shown in Figure 6. It was found that the mercury removal performance of both UiO-66 and UiO-66-Br samples showed an overall enhancement trend with the increasing reaction temperature. This was explained by the fact that a higher adsorption temperature facilitated the enhancement of the interaction force on Hg^0^ during the MOF material removal process, lowered the energy barrier, and ultimately enhanced the reaction system’s overall chemisorption performance. Moreover, for the UiO-66-Br sample, the total amount of Hg^0^ removed was more noticeably improved, indicating that elemental bromine can additionally provide the chemisorption active sites required for the binding of Hg^0^; additionally, the Br functional group’s strong oxidizability makes it favorable for electron trapping and charge transfer, and it has a high electron–hole separation efficiency, which significantly enhances the chemisorption capacity of the sample and significantly enhances the redox capacity of the sample during chemisorption [15]. The Hg^0^-removed cumulative amount of MBC peaked at 150 °C adsorption temperature (139.84 μg/g). Increasing the reaction temperature further hindered Hg^0^ removal from MBC, which was explained by the fact that multilayer adsorption was affected by both chemical and physical adsorption. The former is mainly affected by physical properties such as pore structure, while the latter is primarily influenced by chemical properties such as surface functional groups, and the stability of adsorption products is high. In addition, physical adsorption, as a reversible exothermic process, was mainly accomplished by van der Waals forces between the molecules of monomers of Hg^0^ and biochar, corresponding to a weak bonding force, whereas for chemical adsorption, the adsorption was accomplished by the formation of stable chemical bonds through the electron transfer, exchange, or sharing of electrons with the chemical groups on the surface of the MBC. Therefore, as the adsorption temperature increased further, the physical adsorption of MBC was completely inhibited and destroyed. Additionally, the promotion of the chemical adsorption process was much smaller than the inhibition of the physical adsorption, which led to the gradual weakening of the samples’ overall Hg^0^ adsorption performance. For the UiO-66-Br modified biochar composite adsorbent, when the proportion of MBC was less than or equal to 50%, the samples retained the high-temperature adsorption characteristics of the MOF materials, and the cumulative Hg^0^ removal amount was proportional to the adsorption temperature. The cumulative Hg^0^ removal amount of UiO-66-Br@MBC(9:1 and 5:1) at 250 °C was 4.3 and 1.9 times higher than that at 50 °C, respectively. With lower Hg^0^ removal rates at various adsorption temperatures and nearly complete removal of the inlet Hg^0^ within a 3-h reaction time, close to saturation adsorption, the overall removal performance of the UiO-66-Br@MBC(2:1) samples was better. As a result, increasing the reaction temperature had little effect on the Hg^0^ removal performance. Based on the inherited single-component (MBC and UiO-66-Br) Hg^0^ removal capability and characteristics, the cumulative Hg^0^ removal amount of UiO-66-Br@MBC(1:1) samples in the range of 50~250 °C exceeded 210 μg/g, suggesting that the composite material can expand the adsorption reaction temperature domain and has the potential for optimal Hg^0^ removal. With an adsorption temperature of 150 °C, the effects of adsorption temperature on the three UiO-66-Br@MBC samples (1:2, 1:5, and 1:9) were comparable to those of MBC. Increasing the reaction temperature further would reduce the Hg^0^ removal capacity.

#### 3.2.2. Effect of Composite Ratio

**Figure 7:**
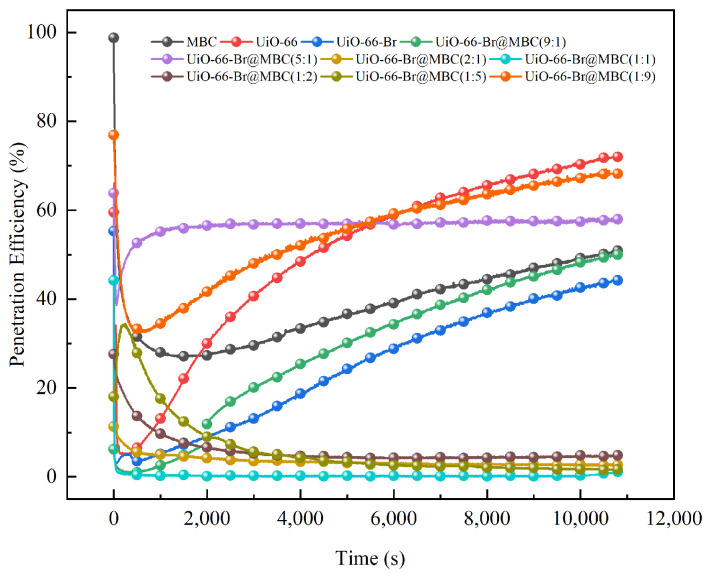
Effect of composite ratio on Hg^0^ removal performance.

For the preparation of UiO-66-Br-based modified biochar composite adsorbent, the effect of composite ratio on the Hg^0^ removal performance is shown in Figure 7. Since it is evident from pervious analyses that composite materials perform well in Hg^0^ removal at 150 °C, this research examines the quantitative correlation mechanism between composite ratios and Hg^0^ removal performance under reaction temperature conditions. In coal-fired power plant operation, the adsorbent injection site is installed in the boiler’s tail flue, and the corresponding flue gas temperature is typically controlled in the range of 120~150 °C.

For MOFs, it was found that the UiO-66 and UiO-66-Br samples had rich surface chemical properties. As a result, the Hg^0^ removal curves at the start of the reaction first rapidly decreased to about 5% and then gradually increased with the removal. Throughout this time, the UiO-66-Br sample’s removal efficiency was consistently lower than the UiO-66 sample’s, confirming the enhancement effect of Br on the removal’s performance. However, the initial MBC removal efficiency was 28%, which, during the reaction process, was essentially in between the modified and unmodified MOF materials. In comparison to single-component UiO-66-Br, the Hg^0^ removal efficiency of UiO-66-Br@MBC(9:1 and 5:1) composites declined when the composite ratio of MBC was smaller. This was explained by the fact that the Fe/Ce metal ions and functional groups in the MBC preferred to bind to the organic ligands and Zr^4+^ in UiO-66-Br during the composite synthesis process utilizing the in situ growth approach. With the formation of a large number of unsaturated coordination metal sites, UiO-66’s own organic framework grows around the carbon chain framework of the MBC and forms a spatial site-blocking effect, which is accompanied by the interruption of the growth evolution process of the MOF material and the formation of the smallest structural unit with MBC. The low proportion of MBC in the composite makes it easy for the MOF material to cover a large area. This prevented the active centers that are helpful for the removal of Hg^0^, such as polymetallic clusters, oxygen vacancies, and carbon skeletons, from being fully exposed and prevented the stimulation of Br groups’ activity. Ultimately, this resulted in the samples performing poorly in Hg^0^ removal. Further increasing the composite ratio of MBC as a substrate carrier allows the MBC to load on the surface of the cross-linked organic framework of the MOFs and provide an abundant pore structure, which in turn prevents the metal oxides from self-polymerizing to form highly agglomerated metal centers on the basis of improving the mass transfer and electron-donor capacity of the entire reaction system [16], thereby improving the Hg^0^ removal performance of the samples. The Hg^0^ removal rate of the prepared materials, particularly for the UiO-66-Br@MBC(2:1 and 1:1) samples, could be consistently maintained at less than 0.5% during the 3-h removal time. This material possessed the highest adsorption capacity (233.22 μg/g) and also quickly reduced the inlet Hg^0^ concentration from 90 μg/m^3^ to approximately 0 μg/m^3^ at the start of the reaction, demonstrating rapid adsorption kinetics. It is characterized by fast adsorption kinetics. Meanwhile, the central metal, Zr^4+^, in the zirconium–oxygen clusters contained in UiO-66 as a MOF material can be a Lewis-acidic active-adsorption site because of its unsaturated coordination property, which is favorable for the removal of Hg^0^ as a Lewis base substance. In addition, during the composite process of MOF materials with biochar modified by Fe and Ce polymetallic doping, on the one hand, the defective metal ions constructed can be introduced into the UiO-66-Br framework nodes and replace some of the Zr^4+^ ions, thus promoting the composites to expose more active sites; on the other hand, the polymetallic ions introduced can also be combined with surrounding C atoms to further stimulate the reactivity of Br functional groups. The number and activity of adsorption/oxidation sites on the surface of the samples were enhanced by mechanisms such as enhancing the charge separation effect and constructing π-complex bonds [17]. Combined with the previous section, it is evident that Br can function as a link between metal ions and organic ligands to facilitate electron transfer during the oxidation process. The catalytic oxidation ability of the samples to Hg^0^ is substantially enhanced, resulting in a low slope for the enhancement of the removal rate during the reaction’s late stages. With the proportion of MBC composite exceeding 50%, the number and activity of reaction sites such as structural defects and constructed oxygen vacancies induced by the high concentration of polymetallic were enhanced. This was conducive to the realization of Hg^0^ removal, accompanied by the enhancement of sample oxidative properties, but with a decrease in the degree of reaction site dispersion. During the adsorption process, it is simple to approach saturation and attain adsorption equilibrium because the pore structure’s abundance as the first adsorption site for absorbing Hg^0^ declines. In the middle and late stages of the reaction, the oxidation site of Hg^0^ on the surface of the sample is oxidized and will be transferred to the neighboring adsorption site of non-catalytic oxidation. As a result, the small amount of pore structure that is present cannot be fitted to the subsequent oxidation reaction that takes place in more reactive sites, causing a significant decrease in the samples’ overall Hg^0^ removal capacity. Four adsorption kinetic models were used to fit the data of mercury removal from the samples under the reaction condition of 150 °C. See Section S6 in the Supplementary Materials for more details on the adsorption kinetics of the samples test.

### 3.3. Hg^0^ Removal Mechanism

#### 3.3.1. Diffusion of Hg^0^ in MOFs

**Figure 8:**
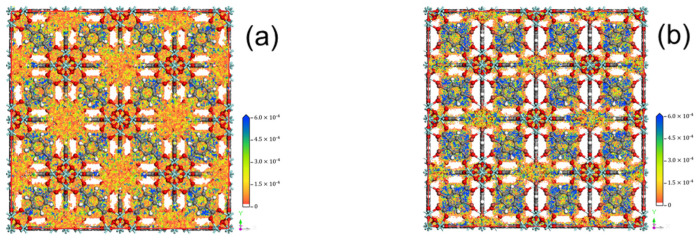
The adsorbed Hg^0^ molecule density from the GCMC simulations:(**a**) UiO-66 and(**b**) UiO-66-Br.

To further reveal the adsorption mechanism of MOFs on Hg^0^, the adsorption behavior process of Hg^0^ in the microporous space of UiO-66, as well as UiO-66-Br, was investigated, in which the density distributions of Hg^0^ under the reaction conditions of 373 K and 100 kPa are shown in Figure 8. The probability distributions of Hg^0^ in the specific spaces of UiO-66 and UiO-66-Br samples show that the adsorption density of the former is more dispersed, while the density points of the UiO-66-Br sample are more concentrated, which, in turn, indicates that the adsorption of Hg^0^ in the structure of the tetrahedral cage is obviously enhanced after the doping modification of the Br element. This is due to the presence of a large number of open metal sites in the UiO-66 cage structure, leading to a large amount of Hg^0^ being adsorbed in the pore cage; for the UiO-66 sample modified by Br functionalization, the Br element in the benzene ring promotes the loss of electrons by Hg^0^, which leads to a strong interaction, resulting in the adsorbed Hg^0^ atom density being significantly higher than that of the UiO-66 sample, and this is also verified by the fact that the density points of Hg^0^ are more concentrated in the tetrahedral cage structure after Br doping. The results obtained in the previous section were also verified.

#### 3.3.2. Elemental Valence

**Figure 9:**
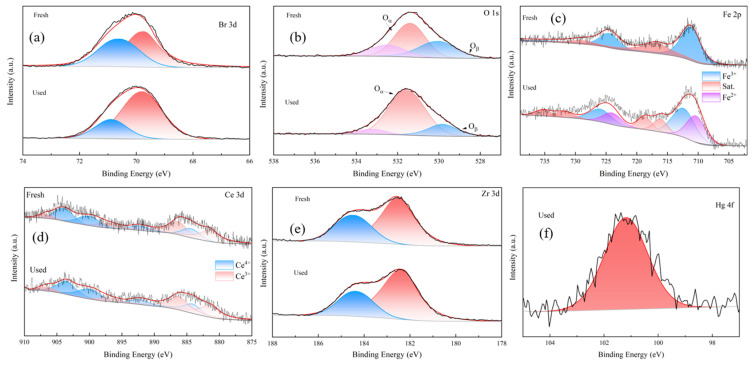
XPS spectra of UiO-66-Br@MBC(1:1) before and after Hg^0^ removal: (**a**) Br 3d, (**b**) O 1s, (**c**) Fe 2p, (**d**) Ce 3d, (**e**) Zr 3d, and (**f**) Hg 4f.

To identify the oxidation sites of UiO-66-Br@MBC(1:1) with optimal Hg^0^ removal performance and to investigate the related reaction mechanism, the elemental valence changes on the surface of the samples before and after the Hg^0^ removal are investigated in this section, and the corresponding XPS spectra are shown in Figure 9. The spectrum of Br 3d (shown in Figure 9a) shows that not only the peak attributed to the Br ion (69.5 eV), but also the peak at 70.5 eV, is attributed to the C-Br covalent bonding. Br, as a halogen element, can be initially oxidized to Hg^0^ by enhancing the charge separation and constructing π-complex bonds with the carbon framework structure. The oxidized mercury was destabilized on the surface of the sample in the form of a [C-Br-Hg-O] complex, accompanied by the one-electron reduction of Br ions. Part of the C-Br bond was broken to form the corresponding unstable radical-Br, corresponding to an increase in the peak area from 32.46% to 95.34%, and coupled with the previously generated complex ligand bond [C-Br-Hg-O] to ultimately generate the HgBr_2_ product and stabilized adsorption on the sample surface.

Three peaks appeared in the O 1s spectrum shown in Figure 9b, and the peak with a binding energy of 530 eV was attributed to the lattice oxygen (O_β_) in the metal oxides, whereas the peaks appearing at 531.4 and 533 eV corresponded to the chemisorbed oxygen (O_α_), which has a strong mobility, and the carboxylic acid oxygen component contained in the sample, respectively. After the Hg^0^ removal, the O_α_/O_β_ ratio on the sample surface increased from 2.31 to 13.3, indicating that O_α_, O_β_, and-Br in an unstable state can act as oxidation sites and participate in the oxidation of Hg^0^ together. The results showed that there was a significant increase in the percentage of chemisorbed oxygen. This could be attributed to the following reasons: first, the lattice oxygen in the metal oxides would be converted into chemisorbed oxygen during the removal process; second, the presence of-Br could stimulate the activation of oxygen species; and third, the large consumption of lattice oxygen during the reaction process, accompanied by a large number of oxygen vacancies constructed, could enhance the electron transfer-enrichment effect of each group on the sample surface, thereby promoting the reorganization of the charge distribution on the surface of the composite adsorbent. Moreover, as the preceding section demonstrated, the sample contains graphite microcrystalline structure. Oxygen vacancies in the formation process will lead to the occurrence of spin-chirp phenomena in the electronic layer of carbon atoms at the edge of the graphite microcrystalline structure around them, increasing the lattice defects on the surface of the samples and enhancing the overall activity of the reaction system, in turn facilitating the catalytic oxidation process of Hg^0^ on the surface of the samples, as well as chemical adsorption [18].

Furthermore, MBC in the composite adsorbent was doped with Fe/Ce at mass fractions of 10% and 4%, respectively; these metal ions with lattice defects will serve as the active sites for chemisorption and take part in the oxidation process of Hg^0^. Among them, according to the Fe 2p spectra (as shown in Figure 9c), it can be found that Fe is mainly endowed in the form of Fe_2_O_3_ on the surface of the samples before Hg^0^ removal, and Fe^2+^ appears on the surface of the samples after the reaction with Fe^3+^/Fe^2+^ = 1.23, which suggests that Fe^3+^ is reduced to Fe^2+^, and the lattice oxygen in the oxides is consumed, accompanied by the oxidation of Hg^0^ and its combination with oxygen species to form a Hg-O-FeO_x−1_ coordination compound. From the Ce 3d spectrum shown in Figure 9d, it can be found that the Ce elements endowed on the surface of the samples before and after Hg^0^ removal are in the state of co-mixing of the two valence species of Ce^4+^/Ce^3+^, indicating that the unsaturated ligand bonds constructed by the Ce elements in the process of removal play the role of the oxidation site and that the ratio of Ce^4+^/Ce^3+^ decreases after removal. The samples contain a large amount of Ce^3+^, which is accompanied by the formation of lattice defects. Due to Ce’s own strong oxygen storage capacity and excellent redox properties, more unsaturated oxygen vacancies with high activity and lattice oxygen with high mobility will continue to be generated during the interconversion of Ce^4+^ and Ce^3+^. These vacancies will be supplemented as the reactive active site for Hg^0^ oxidation [19]. In addition, the redox process can be facilitated by the occurrence of a reaction(Fe^2+^ + Ce^4+^ → Fe^3+^ + Ce^3+^) between Ce, Fe, and their oxides. CeO_2_ can restore lattice oxygen in Fe_2_O_3_ as it is being depleted during the process, which in turn exerts an obvious synergistic promotion effect on the overall Hg^0^ oxidation properties of the samples.

For the important center metal, Zr, in UiO-66, the 3d spectrum (shown in Figure 9e) shows that the peaks attributed to Zr^4+^ appear at 182.5 and 184.8 eV, but the peak shape and position hardly change before and after the removal of Hg^0^, thus indicating that it is difficult to reduce Zr^4+^. Zr^4+^ plays a “catalyst-like” role, though it does not directly take part in the oxidation reaction of Hg^0^. In the composite preparation of UiO-66-Br and MBC, it plays the role of skeleton bridge, which promotes the close combination between the components. Zr^4+^ can promote the metal oxides to be highly dispersed on the surface of the composite material, which facilitates the transfer of electric charge among Fe-O-Zr, Ce-O-Zr, and Fe-O-Ce based on the enhancement of the overall sintering-resistant properties of the samples. This is also accompanied by the production of a significant amount of reactive oxygen species, thus greatly increasing oxygen mobility [20]. The Hg 4f spectrum after Hg^0^ removal is shown in Figure 9f, and it was found that the peak attributed to Hg^2+^ appeared on the surface of the sample (with a binding energy of 101.5 eV), thus verifying that the composite material oxidized the Hg^0^ originally adsorbed on the surface of the sample in the process of removing Hg^0^ and finally produced stable oxidative removal products.

#### 3.3.3. Temperature-Programmed Desorption

**Figure 10:**
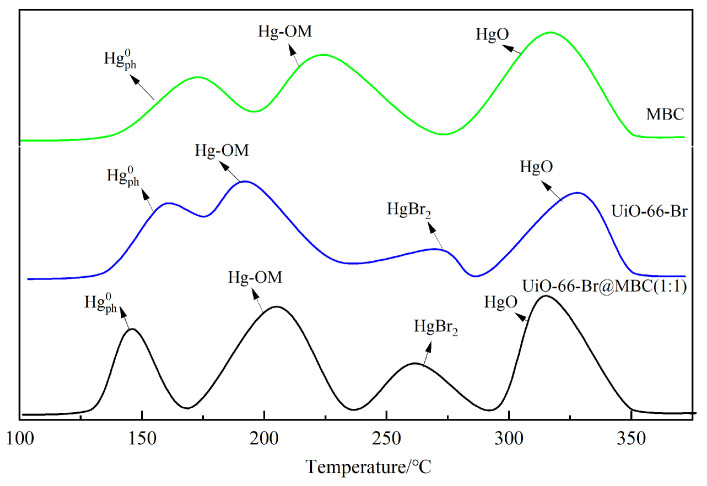
Mercury fugitive form on the surface of samples.

Combined with the previous findings, it was found that the removal of Hg^0^ from the sample was divided into two stages, adsorption and oxidation, in which the adsorption stage of Hg^0^ mainly includes physical and chemical adsorption processes, and the corresponding adsorption products are the physically adsorbed state of mercury(Hg^0^_ph_) and the weakly bonded state of mercury, such as Hg-OM, which is formed through different forms of chemical bonding; subsequently, part of the adsorbed Hg^0^ will undergo oxidation, accompanied by the formation of HgO [21]; eventually, the Hg^0^ removed through different reaction paths coexists in various forms on the sample surface. In this section, to obtain the removal pathways and the corresponding mercury forms, temperature-programmed desorption was carried out on the sample after the removal of Hg^0^, and the results are shown in Figure 10.

With rising temperatures, three independent and obvious desorption peaks with increasing intensities were developed in the MBC desorption curves at 172 °C, 226 °C, and 318 °C, respectively. This was attributed to the strong oxidation of Hg^0^ in the biochar-doped Fe/Ce polymetallic samples, and the main product removed was HgO. Meanwhile, compared with the other samples, due to the simple pore structure, there were fewer micropores and mesopores conducive to the physical adsorption process, and the content of the surface functional groups that promoted the chemical adsorption was low, which corresponded to the lower content of Hg^0^_ph_ and Hg-OM. During the desorption process of the UiO-66-Br, not only the peak of HgBr_2_ desorption appeared near 271 °C, but also the starting desorption temperatures of other kinds of Hg^0^ removal products decreased compared with those of MBC, which was attributed to the well-developed pore structure of the MOFs, which could enhance the internal diffusion rate of the removal products, leading to the early occurrence of the desorption process. Furthermore, the doping of Br increases the ability of electrons to transfer between the metal ions and organic ligands in UiO-66 to improve the charge separation effect while producing HgBr_2_. This results in the formation of more weakly bound Hg-OM, which overlaps most of the desorption region of HgBr_2_ covered by Hg-OM and forms a broader and slower peak domain. Moreover, due to the large specific surface area provided by the organic framework structure, the desorption amounts of Hg^0^_ph_ and Hg-OM were increased compared with those of MBC, and the corresponding desorption areas of the two samples overlapped during the desorption process, resulting in the formation of narrower shoulder peaks. Since the central metal, Zr^4+^, in the zirconium–oxygen cluster is not sufficiently exposed, the HgO formed is mainly generated by the reoxidation of Hg-OM, and the corresponding desorption amount is lower than that of MBC, and no overlapping region is formed with HgBr_2_. After the composite of UiO-66-Br and MBC, the shape of the desorption curve and the position of the desorption peak were similar to that of UiO-66-Br, except that the intensity of the desorption peak and the onset temperature of the desorption were further enhanced and advanced, respectively, with the formation of an obvious peak of desorption of Hg^0^_ph_ at 147 °C, and the Hg-OM and HgO as the major forms of the desorbed Hg^0^. At the same time, the overlap of Hg^0^_ph_, Hg-OM, HgBr_2_, and HgO desorption regions with each other is more significant, and all of them form three broader and slower shoulder peaks. From the previous section, it can be seen that the composite ratio condition can inhibit the agglomeration of metal oxides on the sample surface and form new pore structures and rich surface chemical properties, accompanied by the full exposure of the central active ion Zr^4+^. The Fe and Ce metal ions introduced by MBC can effectively regulate the ratio of adsorption/oxidation sites, thus promoting the adsorption process of Hg^0^, and the oxidative performance is also greatly improved. In addition, the enhanced HgBr_2_ fugitive content led to the highlighting of the HgBr_2_ desorption peaks in UiO-66-Br that were originally covered by the desorption region formed by Hg-OM, which is due to the fact that the carbon framework in UiO-66 as a material for MOFs can stimulate the activity of the C-Br covalent group through the construction of the π-complex bonds, as shown in the previous section [22].

#### 3.3.4. Hg^0^ Removal Mechanism

Based on the comprehensive study of the constitutive relationship between the physicochemical properties of the samples and the performance of mercury removal, combined with the adsorption kinetic fitting and the results of the temperature-programmed desorption, the corresponding Hg^0^ removal mechanism was investigated, and the deep-seated differentiation mechanism between the oxidation and adsorption processes of mercury by the UiO-66-Br-based modified biochar composite adsorbent was revealed. The removal of Hg^0^ by the composite material is affected by the coupling of multiple factors, such as its own physical structure, surface chemical properties, and diffusion processes inside and outside the particles, and is mainly divided into two stages: adsorption and oxidation. At the early stage of the reaction, gaseous Hg^0^ can be captured on the sample surface by the micropores in the pores of the composites and the mesopores of smaller pore sizes in the form of physical adsorption by external diffusion, whereas chemical adsorption occurs mainly through two kinds of adsorption sites. The Lewis acidic active adsorption site provided by unsaturated Zr^4+^ in UiO-66-Br can generate weak chemical bonds with Hg^0^ as a Lewis base; the rich oxygen-containing functional groups contained in the composite material also act as chemical adsorption sites that can be complexed with Hg^0^ and thus endowed on the surface of the samples in the form of organic mercury(Hg-OM). As the reaction proceeds, the diffusion of Hg^0^ inside the pore becomes a rate-controlled step for the Hg^0^ removal from the composite material, while the Hg^0^ adsorbed on the sample surface in the early stage of the subsequent oxidation process, the polymetallic ions, the halogen Br elements, the lattice oxygen, and the chemisorbed oxygen play the role of oxidation sites and form Hg^2+^, as shown in Equations (5)–(11). During this period, metal oxides not only provide lattice oxygen and promote its conversion to chemisorbed oxygen; a reaction between Fe/Ce can also occur which plays a synergistic role in promoting the oxidation of Hg^0^. The presence of the halogenated element Br promotes the deposition of part of the oxidized Hg^0^ on the sample surface in the form of C-Br-Hg-O, resulting in the formation of HgBr_2_. In addition, the element Zr acts as a catalyst-like agent with bridging, dispersive, and mobile properties.

(5) $${\mathrm{Hg}}^{0}(g)\to {\mathrm{Hg}}^{0}(\mathrm{ads})$$

(6) $$2{\mathrm{CeO}}_{2}\to {\mathrm{Ce}}_{2}O_{3}+O_{\beta }$$

(7) $${\mathrm{Fe}}_{2}O_{3}\to 2{\mathrm{FeO}+O}_{\beta }$$

(8) $${2\mathrm{FeO}+2\mathrm{CeO}}_{2}\to {\mathrm{Fe}}_{2}O_{3}+{\mathrm{Ce}}_{2}O_{3}$$

(9) $$O_{\beta }\to O_{\alpha }$$

(10) $${\mathrm{Hg}}^{0}{(\mathrm{ads})+O}_{\alpha }{/O}_{\beta }\to \mathrm{HgO}$$

(11) $${\mathrm{Hg}}^{2+}+{2\mathrm{Br}}^{-}\to {\mathrm{HgBr}}_{2}$$

## 4. Conclusions

1. For the UiO-66-Br@MBC composite samples with different composite ratios obtained by the in situ growth method, the Hg^0^ removal performance was significantly improved, and the two materials could strongly interact with each other, achieving the directional configuration of adsorption sites and oxidation sites while having excellent thermal stability. Among them, UiO-66-Br@MBC(1:1), as the optimal sample, has a rich pore structure with the highest ratio of mesopores to micropores and excellent chemical properties on the surface, which corresponds to a stable Hg^0^ removal rate of about 0.5% in 3 h and a cumulative removal of up to 233.22 μg/g.
2. The oxygen-containing functional groups in MBC can combine with the unsaturated Zr^4+^ in the MOFs material during the composite process, and the introduced metal ions can also form new metal–liganded hydroxyl functional groups with those in the organic ligand, thus realizing the anchoring of UiO-66-Br on the surface of MBC. As a carrier, MBC gave full play to the role of the carrier active component, and the two together constructed a large number of Hg^0^ adsorption/oxidation active sites, which promoted the samples to synergistically remove Hg^0^ substantially on the basis of improving the oxygen-uptake-and-release cycling ability of the metal oxides.
3. The removal of Hg^0^ by the composites is divided into two stages: adsorption and oxidation. The physical adsorption sites are the micropores and smaller pore size mesopores in the pores, while the unsaturated central metal and abundant oxygen-containing functional groups on the zirconium–oxygen clusters act as the chemical adsorption sites. The metal ions, lattice oxygen, chemisorbed oxygen, and-Br introduced during the composite process as the main oxidation sites, together with the adsorption sites, form a stable removal reaction system on the surface of the composite UiO-66-Br@MBC.

## Figures and Tables

**Table 1:** Pore structure parameters of samples.

Sample	BET Specific Surface Area (m^2^/g)	Pore Volume (cm^3^/g)	Average Pore Size (nm)	Relative Specific Pore Volume (%)		
				Microporous	Mesoporous	Macroporous
MBC	107.76	0.113	4.17	30.24	68.68	1.08
UiO-66	1083.52	0.573	2.33	41.70	57.42	0.88
UiO-66-Br	834.78	0.155	2.25	44.14	55.47	0.39
UiO-66-Br@MBC(9:1)	847.96	0.158	2.31	34.61	64.75	0.64
UiO-66-Br@MBC(5:1)	866.17	0.161	2.65	43.95	55.39	0.66
UiO-66-Br@MBC(2:1)	908.08	0.162	2.84	36.30	63.50	0.20
UiO-66-Br@MBC(1:1)	989.89	0.167	2.93	26.43	73.49	0.08
UiO-66-Br@MBC(1:2)	706.72	0.155	3.27	16.32	83.36	0.33
UiO-66-Br@MBC(1:5)	460.19	0.148	3.35	21.69	77.46	0.85
UiO-66-Br@MBC(1:9)	254.61	0.133	3.86	31.73	67.24	1.03

## Data Availability

The data that support the findings of this study are available from the corresponding author upon reasonable request.

## Supplementary Materials

The following supporting information can be downloaded at https://www.mdpi.com/article/10.3390/polym16172508/s1.

## References

[1] Jang H.-N., Back S.-K., Sung J.-H., Kang Y.-S., Jurng J., Seo Y.-C. (2018). The Simultaneous Capture of Mercury and Fine Particles by Hybrid Filter with Powder Activated Carbon Injection. Environ. Pollut..

[2] Li B., Zhang Q.-L., Zhou C. (2016). Effect of Flue Gas Purification Facilities of Coal-Fired Power Plants on Mercury Emission. Proceedings of the 2016 International Conference on Energy Development and Environmental Protection (EDEP 2016).

[3] Winarta J., Shan B., Mcintyre S.M., Ye L., Wang C., Liu J., Mu B. (2020). A Decade of UiO-66 Research: A Historic Review of Dynamic Structure, Synthesis Mechanisms, and Characterization Techniques of an Archetypal Metal-Organic Framework. Cryst. Growth Des..

[4] Zhao S., Huang W., Xie J., Liu W., Qu Z., Yan N. (2021). Mercury Removal from Flue Gas Using UiO-66-Type Metal-Organic Frameworks Grafted with Organic Functionalities. Fuel.

[5] Zhao S., Chen D., Xu H., Mei J., Qu Z., Liu P., Cui Y., Yan N. (2018). Combined Effects of Ag and UiO-66 for Removal of Elemental Mercury from Flue Gas. Chemosphere.

[6] Zhang H.-M., Wang Y.-L., Zhu X.-F., Huang Z.-Z., Pang D.-D., Wang K., Wang C.-H., Song Z.-X., Yin S.-Q., Chang L.-L. (2024). Application of UiO-66 and Its Composites for Remediation and Resource Recovery of Typical Environmental Contaminants: A Review. Rare Met..

[7] Marczak-Grzesik M., Budzyn S., Tora B., Szufa S., Kogut K., Burmistrz P. (2021). Low-Cost Organic Adsorbents for Elemental Mercury Removal from Lignite Flue Gas. Energies.

[8] Gong R., Chen H., Rao X., Chi J., Zhang Z., Xu G., Chen D., Zhou Q., Lu P. (2024). Synthesis of Mn/Cu Co-Doping Mesoporous Carbon by Template Method for Gas Phase Mercury Removal. Fuel.

[9] Jia L., Yu Y., Li Z., Qin S., Guo J., Zhang Y., Wang J., Zhang J., Fan B., Jin Y. (2021). Study on the Hg Removal Characteristics and Synergistic Mechanism of Iron-Based Modified Biochar Doped with Multiple Metals. Bioresour. Technol..

[10] Li M., Qin K., Mao J., Gong Y., Cao Q., Zhang Y., Duan C., Xiao H. (2024). Post-Synthetic Modification on Metal Organic Frameworks Composite for Efficient Removal and Sensitive Detection of Mercury Ions in Water. Chem. Eng. Sci..

[11] Tian L., Li C., Li Q., Zeng G., Gao Z., Li S., Fan X. (2009). Removal of Elemental Mercury by Activated Carbon Impregnated with CeO_2_. Fuel.

[12] Eltaweil A.S., Elshishini H.M., Ghatass Z.F., Elsubruiti G.M. (2021). Ultra-High Adsorption Capacity and Selective Removal of Congo Red over Aminated Graphene Oxide Modified Mn-Doped UiO-66 MOF. Powder Technol..

[13] Jia L., Fan B., Yao Y., Han F., Huo R., Zhao C., Jin Y. (2018). Study on the Elemental Mercury Adsorption Characteristics and Mechanism of Iron-Based Modified Biochar Materials. Energy Fuels.

[14] Hou X., Bian Y., Jin L., Yang L. (2023). Ceria-Based Oxide Catalysts Supported on Metal-Organic Frameworks: Selective Oxidation of Toluene to CO2 and the Doped Metal-Activity Relationship. Catal. Sci. Technol..

[15] Wang S., Ai Z., Niu X., Yang W., Kang R., Lin Z., Waseem A., Jiao L., Jiang H.-L. (2023). Linker Engineering of Sandwich-Structured Metal-Organic Framework Composites for Optimized Photocatalytic H2 Production. Adv. Mater..

[16] Wang J., Qin J., Yang C., Hu Y. (2023). Effect of Ligand Substitution in UiO-66 Metal-Organic Frameworks on the Photocatalytic Oxidation of Acetaldehyde. Chemosphere.

[17] Mu X., Jiang J., Chao F., Lou Y., Chen J. (2018). Ligand Modification of UiO-66 with an Unusual Visible Light Photocatalytic Behavior for RhB Degradation. Dalton Trans..

[18] Zhang T., Zhang J., Wei S., Xiong Z., Xiao R., Chuai X., Zhao Y. (2023). Effect of Hydrothermal Pretreatment on Mercury Removal Performance of Modified Biochar Prepared from Corn Straw. Fuel.

[19] Zhang Z., Liu J., Wang Z., Zhang J. (2021). Bimetallic Fe-Cu-Based Metal-Organic Frameworks as Efficient Adsorbents for Gaseous Elemental Mercury Removal. Ind. Eng. Chem. Res..

[20] Hai N.D., Nguyen M.B., Tan V.M., Huu N.T., Phuong L.B., Huong P.T.M., Nguyen T.D. (2023). Formation of Structural Defects within Fe-UiO-66 for Effective Adsorption of Arsenic from Water. Int. J. Environ. Sci. Technol..

[21] Li C., Duan Y., Tang H., Zhu C., Li Y., Zheng Y., Liu M. (2018). Study on the Hg Emission and Migration Characteristics in Coal-Fired Power Plant of China with an Ammonia Desulfurization Process. Fuel.

[22] Sang X., Zha Q., Nie X., Liu D., Guo Y., Shi X., Ni C. (2020). Interfacial Growth of Metal-Organic Framework on Carboxyl-Functionalized Carbon Nanotubes for Efficient Dye Adsorption and Separation. Anal. Methods.

