# Supplementary material for: A Novel Composite Material UiO-66-Br@MBC for Mercury Removal from Flue Gas: Preparation and Mechanism
Source: Polymers (Basel). 2024 Sep 3;16(17):2508. doi: 10.3390/polym16172508 (PMC11398020; doi:10.3390/polym16172508)
Supplement: Supplementary file 1 [file polymers-16-02508-s001.zip › polymers-3145615-supplementary.pdf]

# Supplementary Materials

## A Novel Composite Material UiO-66-Br@MBC for Mercury Removal from Flue Gas: Preparation and Mechanism

**Zhen Zhang [1], Zikuo Li [1], Youxiang Feng [1], Jingxiang Yu [1], Xikai Zhang [1], Jinchao Wen [1], Haotian Nie [1], Yue Yu [2] and Li Jia [1,*]**

[1] College of Electrical and Power Engineering, Taiyuan University of Technology, Taiyuan 030024, China; tyutzhangzhen@163.com(Z.Z.); 19834008532@163.com(Z.L.); 15734969860@163.com(Y.F.); jingxiangyu0819@163.com(J.Y.); zxklanyi001@163.com(X.Z.); 18835003405@163.com(J.W.); niehaotian0327@163.com (H.N.)

[2] College of Economics and Management, Taiyuan University of Technology, Taiyuan 030024, China; yuyue@tyut.edu.cn

* Correspondence: jiali@tyut.edu.cn

### List of Supplementary Materials

S1. The selection of walnut shells and particle size

S2. Preparation conditions of samples

S3. Preparation of Modified Biochar

S4. Error elimination

S5. Total mercury balance calculations

S6. Adsorption kinetics

S7. ICP elemental analysis of samples

S8. Nitrogen adsorption–desorption isotherms and BJH pore size distribution plot of samples

S9. The PXRD before and after $Hg^0$ removal of samples

**S1. The selection of walnut shells and particle size**

We have explained why walnut shells were selected as the research object, and commented on the selection of particle size in the original text, as follows:

(1) The selection of walnut shells as the research object: The average mercury content of China's coal is 0.15 mg/kg, while Shanxi Province has abundant coal resources, a huge number of coal-fired power plants, and a variety of coal types. The mercury content in Shanxi coal is higher than the national average of 0.22 mg/kg. China has required the mercury concentration limit in the flue gas of coal-fired power plants to be 30 μg/$m^3$ since January 1, 2015. The biomass resources in Shanxi Province are relatively abundant, including walnut shells, corn cobs, and cotton stalks, among which the walnut production ranks second in the country. The utilization of biomass resources was undoubtedly a green and low-carbon effort; the exploration of low-cost coking processes and adsorption processes was a necessary prerequisite for the use of biochar.

We selected walnut shells (WS), corn cobs (CC), cotton stalks (CS) and coconut shells (CH) as raw materials and completed the preparation of different kinds of biochar samples under the same conditions (pyrolysis temperature was 600°C, and pyrolysis time was 10 min). The mercury adsorption characteristics of biochar samples are shown in Figs. S1 and S2. During the 200 min adsorption time, the mercury adsorption performance of WS was the strongest; the initial penetration rate and the final penetration rate were the lowest, which were 6.67% and 66.67%, respectively. In addition, the total amount of mercury adsorbed per unit mass (q) of WS was 2029 ng/g, CH was 724 ng/g, CC was 767 ng/g, and CS was 1102 ng/g. Based on these results, walnut shells were selected as the raw material for the study.

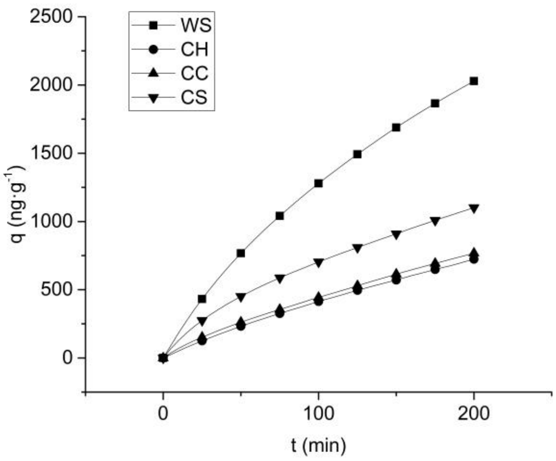


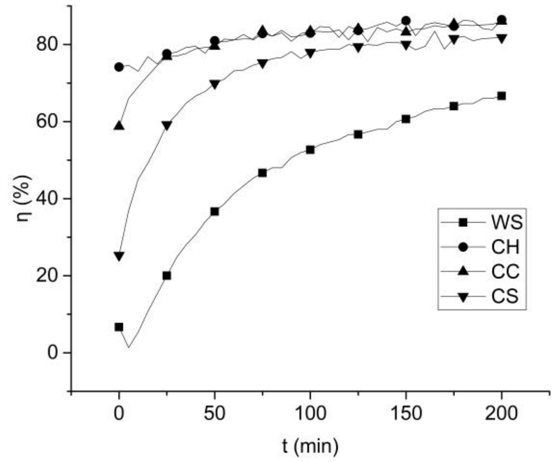


**Figure S1 The total amount of mercury adsorbed per unit mass of biochar**

**Figure S2 The mercury adsorption penetration coefficient of biochar**

(2) The selection of particle size: However, few studies on the effects of different particle sizes on the mercury adsorption characteristics of sorbents have been reported. These few studies have been focused on fly ash as the research object. For instance, Huang[S1] considered three different particle size ranges of fly ash and found that the size of fly ash had impact on the mercury transformation, and the conversion ratio of $Hg^{2+}$ to total Hg increased with increasing size of fly ash. However, the pore structures of fly ash with different particle sizes were not analyzed, and fewer particle size ranges were selected (only three ranges).

In order to obtain the most suitable size range for the study, biomass and the corresponding prepared biochar for eight particle size ranges were studied. The walnut shell materials were all from the same production area. In the process of calculating the mass fractions of different particle sizes of biomass, the 200 g sample was obtained by the quartering method from a 1 kg walnut shell sample, and then the particle size classification was performed using a vibrating machine to obtain samples with eight different particle size ranges. These ranges were >425 μm, 270 μm to 425 μm (50 mesh), 150 μm to 270 μm (100 mesh), 106 μm to 150 μm (150 mesh), 75 μm to 106 μm (200 mesh), 58 μm to 75 μm (250 mesh), 48 μm to 58 μm (300 mesh), and < 48 μm. The weights of samples were calculated by electronic balance, in order to obtain the mass fractions of the biomass samples within different particle size ranges. The biomass samples within different particle size ranges were pyrolyzed at 600°C for 10 min. The

measured mercury adsorption penetration coefficients (η) of biochar (120 min adsorption time) were determined by average value obtained through three parallel experiments.

The results are shown in Table S1. The four particle size ranges, 150 μm to 270 μm (100 mesh), 106 μm to 150 μm (150 mesh), 75 μm to 106 μm (200 mesh), and 58 μm to 75 μm (250 mesh), accounted for a mass fraction of 86.44%, and the mercury adsorption characteristics of the corresponding biochar were better. In addition, although the mercury adsorption effects in the particle size ranges of 48-58 μm and <48 μm were relatively higher, the improvement effect was not obvious, and the mass fractions were too low, resulting a lack of practical application economic value due to the high grinding cost required.

In summary, the particle size of 58 μm to 75 μm was selected for study, based on not only the mass distribution but also the corresponding mercury adsorption characteristics and its economy.

**Table S1 Mass fraction of biomass and mercury adsorption penetration coefficients of biochars within the scope of different particle sizes**

| Item | Particle size (μm) | | | | | | | |
|---|---|---|---|---|---|---|---|---|
| | >425 | 270-425 | 150-270 | 106-150 | 75-106 | 58-75 | 48-58 | < 48 |
| Mass fractions of biomass (%) | 3.91 | 3.49 | 31.99 | 24.93 | 15.76 | 13.76 | 2.55 | 3.61 |
| Mercury adsorption penetration coefficients (η) of biochars (%) | 95.66 | 92.77 | 84.77 | 67.33 | 59.33 | 56.67 | 56.43 | 56.42 |

[S1] Huang, H.; Luo, J. Effect of various fly ash compositions on mercury speciation transformation. *J. Proceeding of the Cess.* **2010**, *30*:70-75.

## S2. Preparation conditions of modified biochar

**Table S2 Preparation conditions of modified biochar**

| No. | Preparation conditions | | | | | | | | | Sample name |
|---|---|---|---|---|---|---|---|---|---|---|
| | Pyrolysis mode | Particle size (μm) | Pyrolysis atmosphere | Metal salt reagents and doping amount | Amount of chemical reagents used (g) | | | | | |
| | | | | | $Mn(CH_3COO)_2{\cdot}4H_2O$ | $Ce(NO_3)_3{\cdot}6H_2O$ | $Co(NO_3)_2{\cdot}6H_2O$ | $CuSO_4{\cdot}5H_2O$ | $FeCl_3{\cdot}6H_2O$ | |
| 1 | Isothermal pyrolysis (800°C) | 58-75 | $N_2$ | / | / | / | / | / | / | Biochar |
| 2 | Isothermal pyrolysis (800°C) | 58-75 | $N_2$ | 10% $FeCl_3{\cdot}6H_2O$ | / | / | / | / | 7.25 | 10%FeBC |
| 3 | Isothermal pyrolysis (800°C) | 58-75 | $N_2$ | 10% $FeCl_3{\cdot}6H_2O$ +1%$CuSO_4{\cdot}5H_2O$ | / | / | / | 0.59 | 7.25 | 1%Cu-FeBC |
| 4 | Isothermal pyrolysis (800°C) | 58-75 | $N_2$ | 10% $FeCl_3{\cdot}6H_2O$ +2%$CuSO_4{\cdot}5H_2O$ | / | / | / | 1.17 | 7.25 | 2%Cu-FeBC |
| 5 | Isothermal pyrolysis (800°C) | 58-75 | $N_2$ | 10% $FeCl_3{\cdot}6H_2O$ +3%$CuSO_4{\cdot}5H_2O$ | / | / | / | 1.76 | 7.25 | 3%Cu-FeBC |

| | | | | | | | | | | |
|---|---|---|---|---|---|---|---|---|---|---|
| 6 | Isothermal pyrolysis (800°C) | 58-75 | $N_2$ | 10% $FeCl_3\cdot6H_2O$ +4%$CuSO_4\cdot5H_2O$ | / | / | / | 2.34 | 7.25 | 4%Cu-FeBC |
| 7 | Isothermal pyrolysis (800°C) | 58-75 | $N_2$ | 10% $FeCl_3\cdot6H_2O$ +5%$CuSO_4\cdot5H_2O$ | / | / | / | 2.93 | 7.25 | 5%Cu-FeBC |
| 8 | Isothermal pyrolysis (800°C) | 58-75 | $N_2$ | 10% $FeCl_3\cdot6H_2O$ +6%$CuSO_4\cdot5H_2O$ | / | / | / | 3.51 | 7.25 | 6%Cu-FeBC |
| 9 | Isothermal pyrolysis (800°C) | 58-75 | $N_2$ | 10% $FeCl_3\cdot6H_2O$ +1%$Co(NO_3)_2\cdot6H_2O$ | / | / | 0.74 | / | 7.25 | 1%Co-FeBC |
| 10 | Isothermal pyrolysis (800°C) | 58-75 | $N_2$ | 10% $FeCl_3\cdot6H_2O$ +2%$Co(NO_3)_2\cdot6H_2O$ | / | / | 1.48 | / | 7.25 | 2%Co-FeBC |
| 11 | Isothermal pyrolysis (800°C) | 58-75 | $N_2$ | 10% $FeCl_3\cdot6H_2O$ +3%$Co(NO_3)_2\cdot6H_2O$ | / | / | 2.22 | / | 7.25 | 3%Co-FeBC |
| 12 | Isothermal pyrolysis (800°C) | 58-75 | $N_2$ | 10% $FeCl_3\cdot6H_2O$ +4%$Co(NO_3)_2\cdot6H_2O$ | / | / | 2.96 | / | 7.25 | 4%Co-FeBC |
| 13 | Isothermal pyrolysis (800°C) | 58-75 | $N_2$ | 10% $FeCl_3\cdot6H_2O$ +5%$Co(NO_3)_2\cdot6H_2O$ | / | / | 3.70 | / | 7.25 | 5%Co-FeBC |
| 14 | Isothermal pyrolysis | 58-75 | $N_2$ | 10% $FeCl_3\cdot6H_2O$ +6%$Co(NO_3)_2\cdot6H_2O$ | / | / | 4.44 | / | 7.25 | 6%Co-FeBC |

| | | | | | | | | | | |
|---|---|---|---|---|---|---|---|---|---|---|
| | (800°C) | | | | | | | | | |
| 15 | Isothermal pyrolysis (800°C) | 58-75 | $N_2$ | 10% $FeCl_3 \cdot 6H_2O$ +1%$Ce(NO_3)_3 \cdot 6H_2O$ | / | 0.47 | / | / | 7.25 | 1%Ce-FeBC |
| 16 | Isothermal pyrolysis (800°C) | 58-75 | $N_2$ | 10% $FeCl_3 \cdot 6H_2O$ +2%$Ce(NO_3)_3 \cdot 6H_2O$ | / | 0.93 | / | / | 7.25 | 2%Ce-FeBC |
| 17 | Isothermal pyrolysis (800°C) | 58-75 | $N_2$ | 10% $FeCl_3 \cdot 6H_2O$ +3%$Ce(NO_3)_3 \cdot 6H_2O$ | / | 1.40 | / | / | 7.25 | 3%Ce-FeBC |
| 18 | Isothermal pyrolysis (800°C) | 58-75 | $N_2$ | 10% $FeCl_3 \cdot 6H_2O$ +4%$Ce(NO_3)_3 \cdot 6H_2O$ | / | 1.86 | / | / | 7.25 | 4%Ce-FeBC |
| 19 | Isothermal pyrolysis (800°C) | 58-75 | $N_2$ | 10% $FeCl_3 \cdot 6H_2O$ +5%$Ce(NO_3)_3 \cdot 6H_2O$ | / | 2.33 | / | / | 7.25 | 5%Ce-FeBC |
| 20 | Isothermal pyrolysis (800°C) | 58-75 | $N_2$ | 10% $FeCl_3 \cdot 6H_2O$ +6%$Ce(NO_3)_3 \cdot 6H_2O$ | / | 2.79 | / | / | 7.25 | 6%Ce-FeBC |
| 21 | Isothermal pyrolysis (800°C) | 58-75 | $N_2$ | 10% $FeCl_3 \cdot 6H_2O$ +1%$Mn(CH_3COO)_2 \cdot 4H_2O$ | 0.68 | / | / | / | 7.25 | 1%Mn-FeBC |
| 22 | Isothermal pyrolysis (800°C) | 58-75 | $N_2$ | 10% $FeCl_3 \cdot 6H_2O$ +2%$Mn(CH_3COO)_2 \cdot 4H_2O$ | 1.34 | / | / | / | 7.25 | 2%Mn-FeBC |

| | | | | | | | | | | |
|---|---|---|---|---|---|---|---|---|---|---|
| 23 | Isothermal pyrolysis (800°C) | 58-75 | $N_2$ | 10% $FeCl_3 \cdot 6H_2O$ +3%$Mn(CH_3COO)_2 \cdot 4H_2O$ | 2.00 | / | / | / | 7.25 | 3%Mn-FeBC |
| 24 | Isothermal pyrolysis (800°C) | 58-75 | $N_2$ | 10% $FeCl_3 \cdot 6H_2O$ +4%$Mn(CH_3COO)_2 \cdot 4H_2O$ | 2.67 | / | / | / | 7.25 | 4%Mn-FeBC |
| 25 | Isothermal pyrolysis (800°C) | 58-75 | $N_2$ | 10% $FeCl_3 \cdot 6H_2O$ +5%$Mn(CH_3COO)_2 \cdot 4H_2O$ | 3.34 | / | / | / | 7.25 | 5%Mn-FeBC |
| 26 | Isothermal pyrolysis (800°C) | 58-75 | $N_2$ | 10% $FeCl_3 \cdot 6H_2O$ +6%$Mn(CH_3COO)_2 \cdot 4H_2O$ | 4.01 | / | / | / | 7.25 | 6%Mn-FeBC |

## S3. Preparation of Modified Biochar

Walnut shell biochar (FeCe/BC) modified by 10%Fe-4%Ce was prepared by a sol–gel combined with co-precipitation method. Walnut shells were ground and sieved to obtain walnut shell biomass with a particle size of 250 mesh (58–75 μm), a weight of 15 g biomass, 8.643 g $FeCl_3·6H_2O$ and 2.214 g $Ce(NO_3)_3·6H_2O$. The mass of the metal compound is obtained from Equation (S1):

$$m_{\text{compound}}=\frac{15}{0.84}\times A\%\times\frac{M_{\text{compound}}}{M_{\text{A}}} \quad \text{(S1)}$$

where $m_{\text{compound}}$ is the compound mass of required element A, $A\%$ is the doping mass ratio of element A and $M_{\text{A}}$ and $M_{\text{compound}}$ are the molar masses of element A and the compound of A, respectively.

The above biomass and metal compounds were dissolved in a mixed solution of 100 mL anhydrous ethanol and 20 mL deionized water and stirred well. Then, 15 mL 1,2 epichlorohydrin and 1 mL DMF were added to form a sol, and after heating in a water bath at 40 °C for 24 h, 2.8 mL ethyl orthosilicate mixed with 0.7 mL anhydrous ethanol was added to the sol to disperse the sol as a wet gel. The precursor material (FeCe/precursor) was obtained by heating it in a water bath at 60 °C for 24 h and then drying and grinding at 70 °C. FeCe/precursor (6–7 g) was weighed and heated at 800 °C for 10 min under $N_2$ airflow with a flow rate of 200 mL/min to obtain FeCe/BC. In addition, unmodified BC was obtained by pyrolysis of 250 mesh walnut shell biomass under the same conditions.

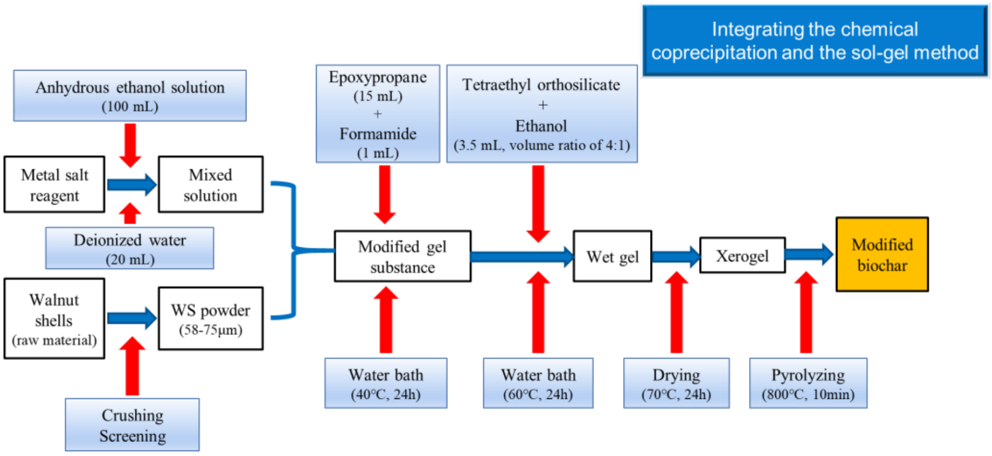


**Figure S3 Flowchart of the sample's preparation process**

## S4. Error elimination

This paper focused on the adsorption characteristics of $Hg^0$, so in order to eliminate the corresponding interference in the experimental process, before the experimental gas entered the VM3000, the $SnCl_2$ solution was used to reduce the $Hg^{2+}$ (some $Hg^0$ oxidized on the surface of samples and escaped) in the experimental gas to $Hg^0$, and then into the VM3000 with the original $Hg^0$ in the flue gas, thus the adsorption characteristics on $Hg^0$ were obtained. As a result, the sorbent performance parameters are not exaggerated.

Moreover, combining with the results obtained by TPD, it is found that there is some elemental mercury oxidized on the surface of the samples. In order to verify this oxidized elemental mercury on the surface of samples escaped, a comparative experiment was performed (using $SnCl_2$ solution and not using $SnCl_2$ solution before the flue gas entered the VM3000) under the same adsorption conditions. The results are shown in Fig. S4. The values detected with the former was more than with the latter. Therefore, this mercury may have escaped during the adsorption process.

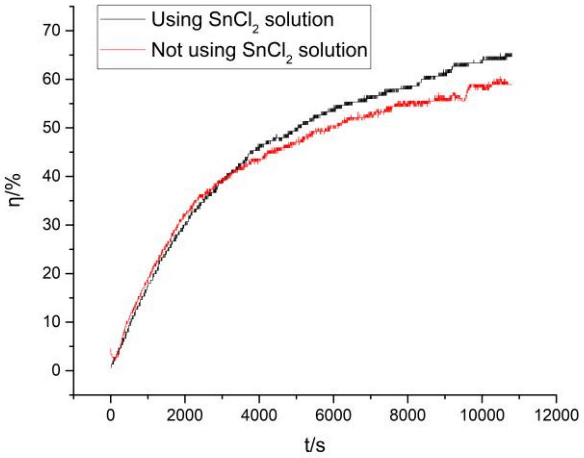


**Figure S4 The results of comparative experiment**

Additionally, this elemental mercury (oxidized on the surface of the samples and escaped) was as the outlet mercury in the mercury balance calculation.

## S5. Total mercury balance calculations

In this paper, the total mercury balance calculations were performed for both the mercury adsorption and the TPD experiments.

The mercury mass balance rate is generally the ratio of the mass of mercury at the outlet to the mass of mercury at the inlet of the experimental system. The calculation method is shown in Equation. (S2):

$$R = \frac{m_{Hg,output}}{m_{Hg,input}} \times 100\% \quad \text{(S2)}$$

where $m_{Hg,\ input}$ is the total mercury content at the inlet of the mercury adsorption experiment on the fixed bed during the adsorption time, which is the inlet mass of $Hg^0$ in the simulated flue gas, in ng. $m_{Hg,\ output}$ is the total mercury content at the outlet of the mercury adsorption experiment on the fixed bed during the adsorption time, including the mass of $Hg^0$ at the outlet in the flue gas after adsorption, and the total mercury enrichment mass in the samples after adsorption, in ng.

(a) The total mercury balance calculations for the mercury adsorption experiment

The total mercury balance for the mercury adsorption process on the fixed-bed is shown in Table S3. A Lumex mercury analyzer was used to obtain the mercury content of the sample that adsorbed mercury, which was used as the corresponding mercury enrichment mass on the surface of the adsorbent.

**Table S3 The total mercury balance for the mercury adsorption process on the fixed-bed**

| Sorbents | Input $Hg^0$ mass/ng | Output $Hg^0$ mass/ng | Hg mass on sorbents/ng | Hg mass balance/% |
|---|---|---|---|---|
| Biochar | 25200 | 23288 | 1408 | 98 |
| Fe/BC | 25200 | 22421 | 2527 | 99 |
| Fe-1%Cu/BC | 25200 | 19475 | 6733 | 104 |
| Fe-2%Cu/BC | 25200 | 11254 | 14450 | 102 |
| Fe-3%Cu/BC | 25200 | 16411 | 10049 | 105 |
| Fe-4%Cu/BC | 25200 | 17125 | 12359 | 117 |
| Fe-5%Cu/BC | 25200 | 18707 | 7753 | 105 |
| Fe-6%Cu/BC | 25200 | 19701 | 9531 | 116 |
| Fe-1%Co/BC | 25200 | 21534 | 8454 | 119 |
| Fe-2%Co/BC | 25200 | 21424 | 8816 | 120 |

| | | | | |
|---|---|---|---|---|
| Fe-3%Co/BC | 25200 | 14902 | 11306 | 104 |
| Fe-4%Co/BC | 25200 | 17071 | 10397 | 109 |
| Fe-5%Co/BC | 25200 | 20037 | 8691 | 114 |
| Fe-6%Co/BC | 25200 | 20368 | 2060 | 89 |
| Fe-1%Ce/BC | 25200 | 13408 | 15068 | 113 |
| Fe-2%Ce/BC | 25200 | 16944 | 11532 | 113 |
| Fe-3%Ce/BC | 25200 | 20555 | 9433 | 119 |
| Fe-4%Ce/BC | 25200 | 20577 | 8151 | 114 |
| Fe-5%Ce/BC | 25200 | 21608 | 7120 | 114 |
| Fe-6%Ce/BC | 25200 | 21781 | 2915 | 98 |
| Fe-1%Mn/BC | 25200 | 18709 | 10775 | 117 |
| Fe-2%Mn/BC | 25200 | 8307 | 16893 | 100 |
| Fe-3%Mn/BC | 25200 | 14674 | 13802 | 113 |
| Fe-4%Mn/BC | 25200 | 16087 | 13397 | 117 |
| Fe-5%Mn/BC | 25200 | 17616 | 9348 | 107 |
| Fe-6%Mn/BC | 25200 | 19816 | 2612 | 89 |

(b) The total mercury balance calculations for mercury adsorption in the TPD experiment

The mercury enrichment mass of sorbent can be determined from integrating the results of the desorption curve during the desorption time, and the mercury mass balance rate can be calculated by combining the inlet mercury mass and the outlet mercury mass on the fixed-bed during the corresponding adsorption process. The total mercury balance results are shown in Table S4.

**Table S4 The total mercury balance for the mercury desorption process on the fixed-bed**

| Sorbents | Input $Hg^0$ mass/ng | Output $Hg^0$ mass/ng | Hg mass on sorbents/ng | Hg mass balance/% |
|---|---|---|---|---|
| Biochar | 25200 | 23288 | 1660 | 99 |
| Fe/BC | 25200 | 22421 | 5551 | 111 |
| Fe-1%Cu/BC | 25200 | 19475 | 4465 | 95 |
| Fe-2%Cu/BC | 25200 | 11254 | 17474 | 114 |
| Fe-3%Cu/BC | 25200 | 16411 | 8537 | 99 |
| Fe-4%Cu/BC | 25200 | 17125 | 7319 | 97 |
| Fe-5%Cu/BC | 25200 | 18707 | 7501 | 104 |

| | | | | |
|---|---|---|---|---|
| Fe-6%Cu/BC | 25200 | 19701 | 3483 | 92 |
| Fe-1%Co/BC | 25200 | 21534 | 5430 | 107 |
| Fe-2%Co/BC | 25200 | 21424 | 2012 | 93 |
| Fe-3%Co/BC | 25200 | 14902 | 11558 | 105 |
| Fe-4%Co/BC | 25200 | 17071 | 8129 | 100 |
| Fe-5%Co/BC | 25200 | 20037 | 4911 | 99 |
| Fe-6%Co/BC | 25200 | 20368 | 5336 | 102 |
| Fe-1%Ce/BC | 25200 | 13408 | 11792 | 100 |
| Fe-2%Ce/BC | 25200 | 16944 | 7248 | 96 |
| Fe-3%Ce/BC | 25200 | 20555 | 2125 | 90 |
| Fe-4%Ce/BC | 25200 | 20577 | 9159 | 118 |
| Fe-5%Ce/BC | 25200 | 21608 | 6868 | 113 |
| Fe-6%Ce/BC | 25200 | 21781 | 7451 | 116 |
| Fe-1%Mn/BC | 25200 | 18709 | 9767 | 113 |
| Fe-2%Mn/BC | 25200 | 8307 | 18153 | 105 |
| Fe-3%Mn/BC | 25200 | 14674 | 13298 | 111 |
| Fe-4%Mn/BC | 25200 | 16087 | 7097 | 92 |
| Fe-5%Mn/BC | 25200 | 17616 | 9096 | 106 |
| Fe-6%Mn/BC | 25200 | 19816 | 3620 | 93 |

Moreover, the error in the mercury balance calculation is affected by many factors, including the velocity fluctuation of the simulated flue gas. It is difficult to accurately calculate the error caused by each factor. Generally, it is believed that the mercury balance rate is in the range of 70% to 130%, indicating that the experimental results are accurate.

All mercury balances in this paper are in the range of 87% to 121%, indicating that the experimental results are reliable.

## S6. Adsorption kinetics

Since the adsorption of $Hg^0$ by mercury adsorbent is mainly classified into external and internal mass transfer processes, which contain both physical and chemical adsorption effects. In this work, four adsorption kinetic models, namely, the quasi-first order kinetic model, the quasi-second order kinetic model, the intra-particle diffusion model, and the Elovich model, were used to fit the data of mercury removal from the samples under the reaction condition of 150 °C, to study the reaction mechanism and to determine the rate-controlling steps in the adsorption process. To identify the adsorption sites, the proportion of physical and chemical adsorption in the adsorption process was further clarified. Among them, the quasi-first order kinetic model and the intra-particle diffusion model mainly study the physical adsorption process, while the quasi-second order kinetic model and the Elovich model focus on the chemical adsorption. The equations of the four models are shown in equations. (S3)-(S6), and the fitted parameters of adsorption kinetics under the condition of adsorption temperature of 150 °C are shown in Table S5.

Quasi-first order kinetic model:

$$q = q_e(1 - e^{-tk_1}) \tag{S3}$$

Quasi-second order kinetic model:

$$q = \frac{q_e^2 k_2 t}{1 + q_e k_2 t} \tag{S4}$$

Intra-particle diffusion model:

$$q = k_{id} t^{\frac{1}{2}} + C \tag{S5}$$

Elovich model:

$$q = \frac{1}{\beta}\ln(t + t_0) - \frac{1}{\beta}\ln(t_0) \tag{S6}$$

Where: $q$ is the cumulative $Hg^0$ removal from the sample at time $t$, ng/g; $q_e$ is the equilibrium $Hg^0$ removal, ng/g; $t$ is the adsorption time, s; $k_1$ is the quasi-first order rate constant, $s^{-1}$; $k_2$ is the quasi-second order rate constant, ng/(g·s); $k_{id}$ is the rate constant for intra-particle diffusion, ng/(g·$s^{1/2}$); $C$ is the constant related to the thickness of the

boundary layer, ng/g; $\alpha$ is the initial adsorption rate, ng/(g·s$^{1/2}$); $\beta$ is a constant related to the surface coverage and activation energy, ng/g; $t_0$=1/($\alpha$·$\beta$), s.

**Table S5. Adsorption kinetic fitting parameters of samples**

| Sample | Quasi-first order kinetic model | | | | Quasi-second order kinetic model | | | |
|---|---|---|---|---|---|---|---|---|
| | $R^2$ | $k_1$ | $q_e$ | $\gamma$ | $R^2$ | $k_2$ | $q_e$ | $\gamma$ |
| MBC | 0.9998 | $3.26\times10^{-5}$ | 474071 | 0.2950 | 0.9998 | $1.98\times10^{-11}$ | 885802 | 0.1579 |
| UiO-66 | 0.9998 | $5.80\times10^{-5}$ | 389546 | 0.1029 | 0.9997 | $4.77\times10^{-11}$ | 691945 | 0.0580 |
| UiO-66-Br | 0.9999 | $4.11\times10^{-6}$ | 5315570 | 0.0326 | 0.9999 | $1.97\times10^{-13}$ | 10529078 | 0.0164 |
| UiO-66-Br@MBC(9:1) | 0.9999 | $9.52\times10^{-5}$ | 794537 | 0.1113 | 0.9999 | $4.68\times10^{-12}$ | 1272912 | 0.0695 |
| UiO-66-Br@MBC(5:1) | 0.9999 | $1.02\times10^{-5}$ | 962081 | 0.1049 | 0.9999 | $2.81\times10^{-12}$ | 1872031 | 0.0539 |
| UiO-66-Br@MBC(2:1) | 0.9999 | $3.43\times10^{-6}$ | 5642205 | 0.0377 | 0.9999 | $1.49\times10^{-13}$ | 11388183 | 0.0187 |
| UiO-66-Br@MBC(1:1) | 0.9999 | $6.61\times10^{-7}$ | 34230012 | 0.0068 | 0.9999 | $4.81\times10^{-15}$ | 68598460 | 0.0034 |
| UiO-66-Br@MBC(1:2) | 0.9999 | $8.75\times10^{-6}$ | 2175962 | 0.0986 | 0.9999 | $9.57\times10^{-13}$ | 4461236 | 0.0481 |
| UiO-66-Br@MBC(1:5) | 0.9997 | $2.23\times10^{-5}$ | 774918 | 0.2855 | 0.9997 | $6.35\times10^{-12}$ | 1651035 | 0.1387 |
| UiO-66-Br@MBC(1:9) | 0.9997 | $7.16\times10^{-5}$ | 553271 | 0.2787 | 0.9997 | $2.17\times10^{-12}$ | 1397152 | 0.1104 |

| Sample | Intra-particle diffusion model | | | Elovich model | | |
|---|---|---|---|---|---|---|
| | $R^2$ | $k_{id}$ | $C$ | $R^2$ | $\alpha$ | $\beta$ |
| MBC | 0.9768 | 1656.5 | -40509 | 0.9997 | 15.55 | $2.42\times10^{-6}$ |
| UiO-66 | 0.9839 | 2132.5 | -47902 | 0.9996 | 20.08 | $3.28\times10^{-6}$ |
| UiO-66-Br | 0.9628 | 2674.6 | -69027 | 0.9999 | 21.87 | $1.92\times10^{-7}$ |
| UiO-66-Br@MBC(9:1) | 0.9621 | 1004.6 | -19421 | 0.9999 | 7.36 | $5.16\times10^{-6}$ |
| UiO-66-Br@MBC(5:1) | 0.9629 | 1155.5 | -28779 | 0.9999 | 9.84 | $1.11\times10^{-6}$ |
| UiO-66-Br@MBC(2:1) | 0.9587 | 2457.4 | -64619 | 0.9999 | 19.63 | $2.99\times10^{-7}$ |
| UiO-66-Br@MBC(1:1) | 0.9599 | 2835.5 | -73938 | 1.0000 | 22.70 | $2.98\times10^{-8}$ |
| UiO-66-Br@MBC(1:2) | 0.9570 | 2498.0 | -67150 | 0.9999 | 19.74 | $4.75\times10^{-7}$ |
| UiO-66-Br@MBC(1:5) | 0.9541 | 2447.0 | -68646 | 0.9995 | 18.93 | $1.85\times10^{-6}$ |
| UiO-66-Br@MBC(1:9) | 0.9504 | 2193.4 | -51394 | 0.9995 | 14.27 | $1.17\times10^{-6}$ |

In the table: $\gamma$ is the degree of adsorption reaction, i.e., the ratio of actual removal amount to predicted saturated adsorption amount.

It was found that the correlation coefficients $R^2$ of the quasi-first order and quasi-second order kinetic models as well as the Elovich model for the three adsorption

kinetics exceeded 0.999, which indicated that the three kinetic models were suitable for the removal of $Hg^0$ from UiO-66-Br@MBC composites, and also indicated that the removal of $Hg^0$ from composites was influenced by multiple factors such as the physical structure of the composites, the chemical properties of the surface, and the diffusion process both inside and outside of the particles. Compared with MBC, the $R^2$ values of the composites obtained with different composite ratios were increased, and the $\gamma$ values of the adsorption reaction degree were decreased, which can indicate that the physical/chemical adsorption properties of the samples were greatly improved. In contrast, the fit of the $Hg^0$ removal process using the intra-particle diffusion model was low, with $R^2$ below 0.97 and $C$ value not 0, indicating that the influence of internal diffusion in the pore channels of the composites was relatively small. Therefore, the diffusion process of $Hg^0$ inside the pore channel becomes the rate-controlling step in the $Hg^0$ removal process of the composite. Comparing the predicted equilibrium adsorption amounts obtained from the quasi-first order and quasi-second order kinetic models with the actual adsorption amounts, it can be found that all the samples are far from reaching the saturation adsorption state. Especially when the composite ratio is 1:1, the predicted equilibrium adsorption amount of the composites is much higher than that of other composite ratios and the $\gamma$-values are only 0.0068 and 0.0034, which are consistent with the actual change rule. This confirms that the composites obtained at 1:1 ratio have the most excellent $Hg^0$ removal performance and very high adsorption capacity. By comparing the fitting correlation coefficients and initial adsorption rates of the Elovich model, it can be found that the model fits extremely well and is more suitable for describing the $Hg^0$ removal process of the composites, which is due to the fact that the model considers the influence of the pore structure of the material surface and the mass transfer diffusion process while considering the effect of the surface chemical adsorption. Among them, the UiO-66-Br@MBC(1:1) corresponds to the highest $\alpha$-value due to its excellent surface chemistry, and therefore has a high reaction rate at the initial stage of the reaction.

## S7. ICP elemental analysis of samples

The test results of ICP are shown in Table S2. From the results of the ICP test, it can be seen that although 10% Fe and 4% Ce were added to the biochar during the preparation process, the actual test data of Fe and Ce were only 6.7% and 2.48%. This indicates that some metal ions were not successfully loaded onto the biochar. Similarly in the preparation of MOFs, the content of central metallic zirconium ions was lower than the theoretical value, which indicates that the synthesis rate of MOFs did not reach 100%, and some zirconium ions were lost in the preparation process. As for the composites, it is clear that as the proportion of biochar in them increases the content of Fe and Ce increases, and correspondingly the content of Zr decreases. We have added Theoretical content of samples and ICP-OES test result in the supplementary material as requested.

**Table S6 Theoretical content of samples and ICP-OES test result**

| Sample | Zr Content(wt%) | | Br Content(wt%) | | Fe Content(wt%) | | Ce Content(wt%) | |
|---|---|---|---|---|---|---|---|---|
| | Theoretical | ICP | Theoretical | ICP | Theoretical | ICP | Theoretical | ICP |
| MBC | 0 | 0 | 0 | 0 | 10.00 | 6.70 | 4.00 | 2.48 |
| UiO-66 | 35.56 | 30.23 | 0 | 0 | 0 | 0.00 | 0 | 0.00 |
| UiO-66-Br | 32.70 | 23.87 | 44.50 | 26.70 | 0 | 0.00 | 0 | 0.00 |
| UiO-66-Br@MBC(9:1) | 29.43 | 21.19 | 40.05 | 26.03 | 1.00 | 0.85 | 0.40 | 0.37 |
| UiO-66-Br@MBC(5:1) | 27.25 | 22.07 | 37.08 | 27.07 | 1.67 | 1.54 | 0.67 | 0.61 |
| UiO-66-Br@MBC(2:1) | 21.80 | 16.79 | 29.67 | 22.25 | 3.33 | 2.83 | 1.33 | 1.16 |
| UiO-66-Br@MBC(1:1) | 16.35 | 13.41 | 22.25 | 17.80 | 5.00 | 4.30 | 2.00 | 1.64 |
| UiO-66-Br@MBC(1:2) | 10.90 | 7.74 | 14.83 | 12.46 | 6.67 | 5.14 | 2.67 | 2.11 |
| UiO-66-Br@MBC(1:5) | 5.45 | 4.58 | 7.42 | 6.38 | 8.33 | 6.08 | 3.33 | 2.40 |
| UiO-66-Br@MBC(1:9) | 3.27 | 2.32 | 4.45 | 4.01 | 9.00 | 6.30 | 3.60 | 2.52 |

## S8. Nitrogen adsorption–desorption isotherms and BJH pore size distribution plot of samples

UiO-66 has a large specific surface area and pore volume and rich pore structure, with micropores and smaller pore size mesopores dominating the pore size, possessing a larger $Hg^0$ storage space and more dispersed active adsorption sites. UiO-66 exhibits a typical type I $N_2$ adsorption isotherm, i.e., a rapid increase in adsorption due to continuous filling of micropores occurs in the region of lower relative pressures, and adsorption appears at a saturation value after reaching a certain relative pressure. After Br modification, UiO-66-Br still has the characteristics of type I adsorption isotherm, but the percentage of micropores and mesopores is increased from the pore size distribution, and the micropores and mesopores provide the adsorption sites for monomers of mercury, so it is favourable to the adsorption of $Hg^0$, which also verifies the enhancement of the performance of Br on mercury removal. According to the IUPAC classification, the MBC and UiO-66-Br@MBC samples were a mixture of type I and IV isotherms for the $N_2$ sorption and desorption curves, which indicated that the adsorbents contained both microporous and mesoporous structures. MBC contains a large number of mesopores with the largest average pore size, and its $N_2$ adsorption and desorption curves have typical H4-type hysteresis loops, which may be attributed to its large pore size mesopores or crystal defects. With the increase of biochar composite ratio, the desorption and adsorption curves of the UiO-66-Br@MBC samples began to be incompletely overlapped, accompanied by the appearance of H4-type hysteresis loops without an obvious saturated adsorption plateau, and the width of hysteresis loops increased with the increase of MBC content, which indicated that the pore size distribution of the samples gradually became more homogeneous. This type of hysteresis loop is often found in adsorbent materials with a mixture of microporous and mesoporous pores and solids with narrow slit pores, which is consistent with the pore size distribution results. As can be seen from the pore size distribution graphs, when the MBC composite ratio is lower than 50%, the pore sizes of UiO-66-Br@MBC (9:1, 5:1, 2:1, 1:1) are mainly concentrated around 2~3 nm. With the increase of MBC composite

ratio, the range of pore size distribution gradually becomes wider, and the proportion of large pore size mesopores increases, which is due to the fact that part of UiO-66-Br grows in the pore channels of MBC and forms mesopores during the composite process to achieve the optimisation of pore structure. UiO-66-Br@MBC(1:1) possesses the highest proportion of micropores and mesopores. And when the MBC composite ratio was further increased, the pore structure of UiO-66-Br@MBC samples gradually evolved towards MBC, with the percentage of micropores and small-sized mesopores decreasing and the percentage of macropores increasing.

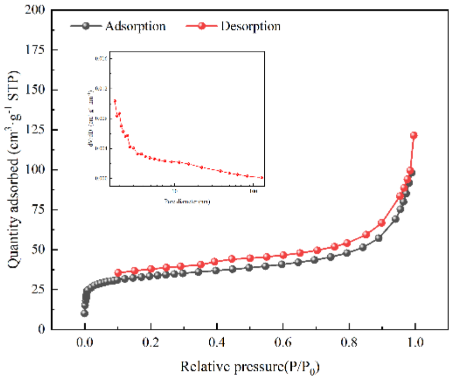


(a) MBC

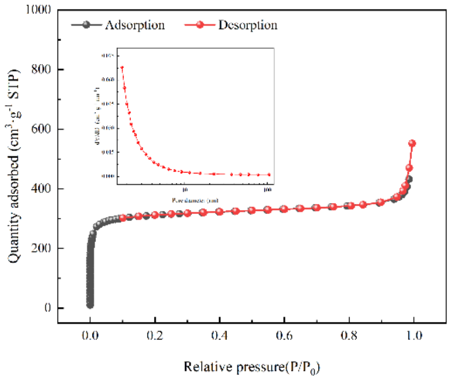


(b) UiO-66

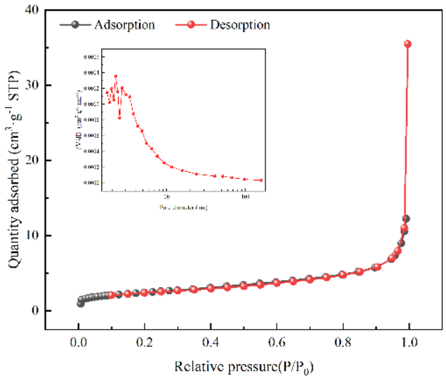


(c) UiO-66-Br

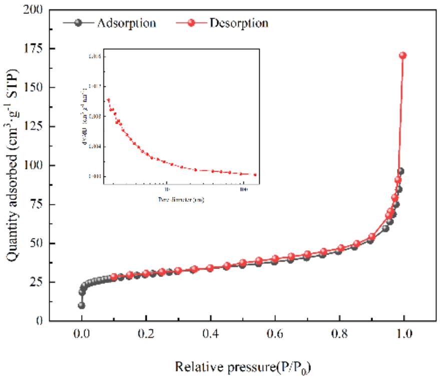


(d) UiO-66-Br@MBC（9:1）

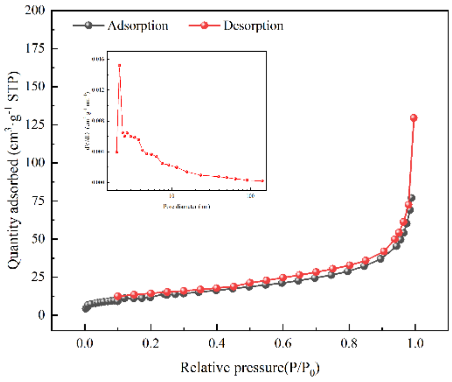


(e) UiO-66-Br@MBC（5:1）

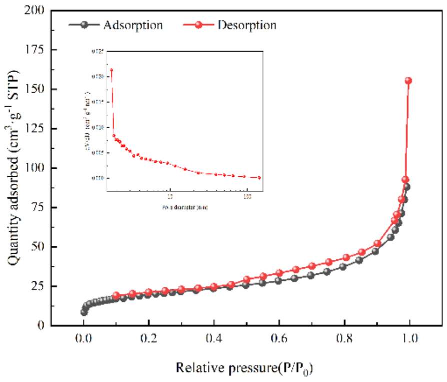


(f) UiO-66-Br@MBC（2:1）

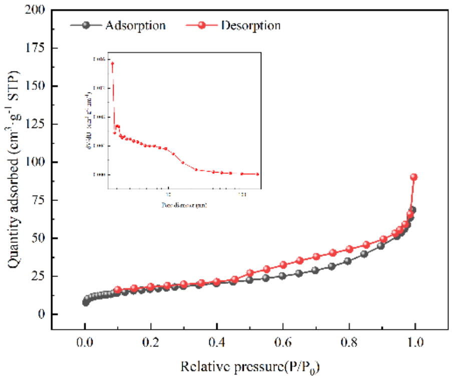


(g) UiO-66-Br@MBC（1:1）

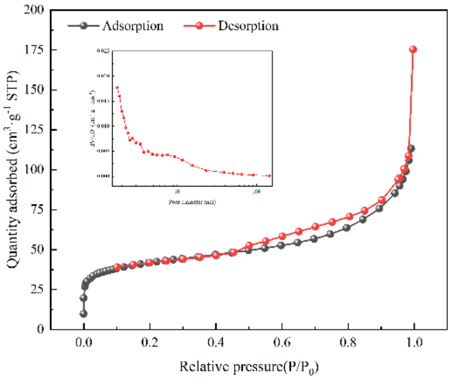


(h) UiO-66-Br@MBC（1:2）

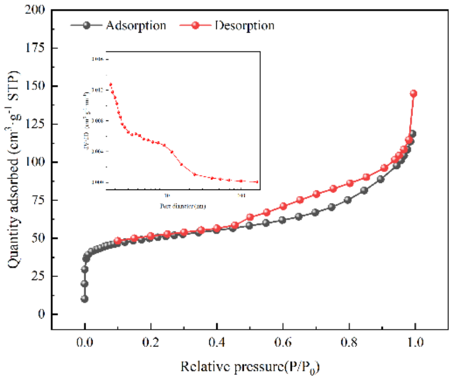


(i) UiO-66-Br@MBC（1:5）

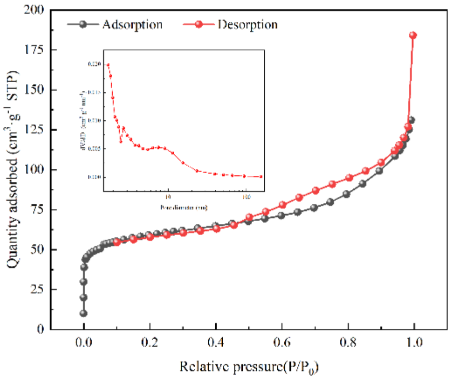


(j) UiO-66-Br@MBC（1:9）

**Figure S5 $N_2$ adsorption–desorption isotherms and BJH pore size distribution plot**

## S9. The PXRD before and after Hg removal of samples

The structural unit of UiO-66 is composed of [$Zr_6O_4(OH)_4$] metal clusters connected with 12 $H_2BDC$ coordination, which is the highest number of organic ligand-metal cluster coordination that can be possessed in MOFs. The dense structural units make the whole structure stably connected; secondly, Zr is highly oxygenophilic, so the stronger Zr-O bond in the UiO-66 structure also increases the stability of the crystal structure. So it can be observed from the Figure S6 that both UiO-66 and UiO-66-Br samples show a slight decrease in peak intensity and an increase in peak broadening after Hg removal, which suggests that the MOFs lead to some degree of structural disorder within the material during the adsorption of $Hg^0$. However, the preservation of the position of the main peak indicates that the overall crystal structure of the two samples remains relatively stable. Moreover, no new peaks appeared in the PXRD spectra of the samples after Hg removal, which proved that the $Hg^0$ was mainly adsorbed on the surface or in the pores of the MOFs, and no new crystal phase was formed. For UiO-66-Br@MBC (9:1, 5:1, 2:1, 1:1), the PXRD of the samples before and after Hg removal only showed a decrease in the intensity of the characteristic peaks attributed to UiO-66, and the positions of the peaks remained basically unchanged. This was explained by the fact that the Fe/Ce metal ions and functional groups in the MBC preferred to bind to the organic ligands and $Zr^{4+}$ in UiO-66-Br during the composite synthesis process utilizing the in-situ growth approach. With the formation of a large number of unsaturated coordination metal sites, UiO-66's own organic framework grows around the carbon chain framework of the MBC. The above results are in agreement with the SEM results. Furthermore, it is evident that the composite adsorbent undergoes a crystallization passivation effect when the composite ratio of MBC is higher than 50%. Due to the presence of metal oxides ($Fe_2O_3$, $Fe_3O_4$, $CeO_2$) in the samples, which can play an oxidising role in the Hg removal process, the intensity of the characteristic peaks corresponding to the metal oxides is weakened, but the characteristic diffraction peaks of the Hg-containing species and other substances do not appear in the graphs, which may be attributed to the small amount of Hg compounds

generated in the adsorbent and the small and well-dispersed particles.

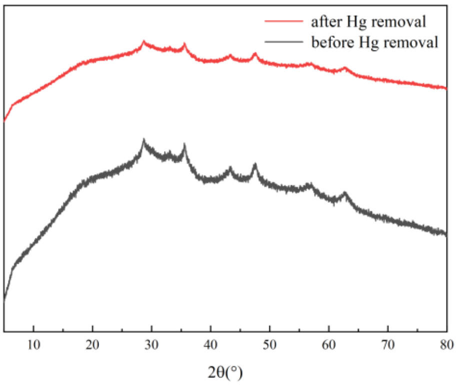


(a) MBC

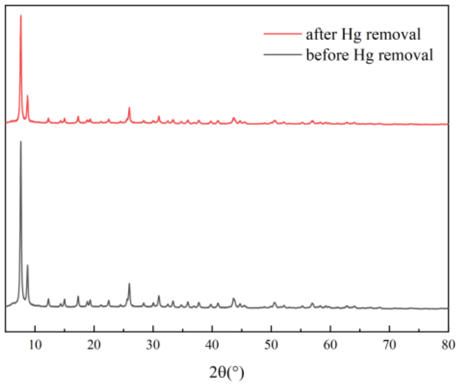


(b) UiO-66

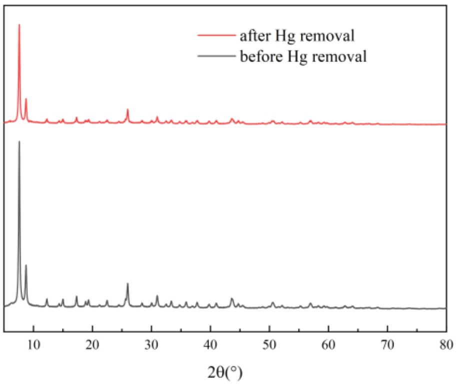


(c) UiO-66-Br

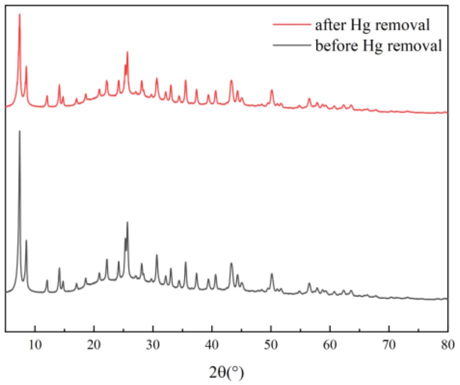


(d) UiO-66-Br@MBC（9:1）

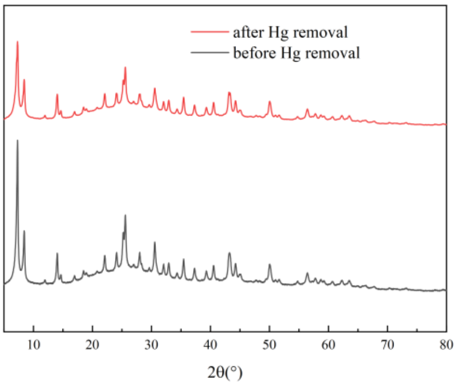


(e) UiO-66-Br@MBC（5:1）

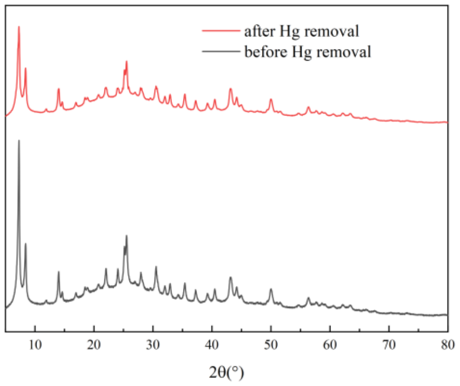


(f) UiO-66-Br@MBC（2:1）

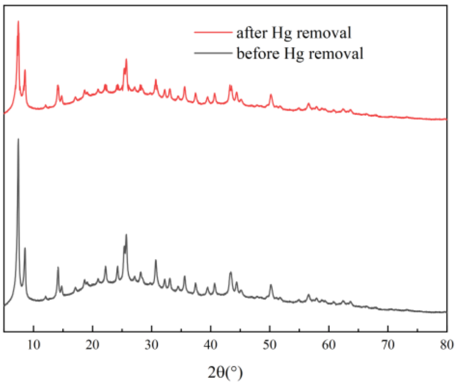


(g) UiO-66-Br@MBC（1:1）

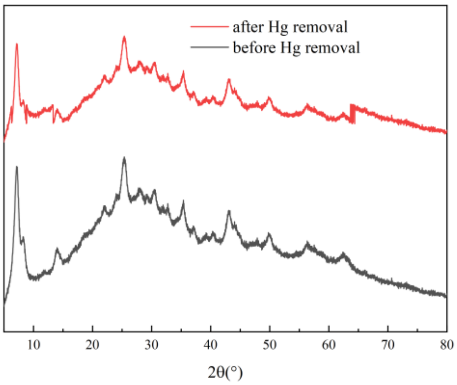


(h) UiO-66-Br@MBC（1:2）

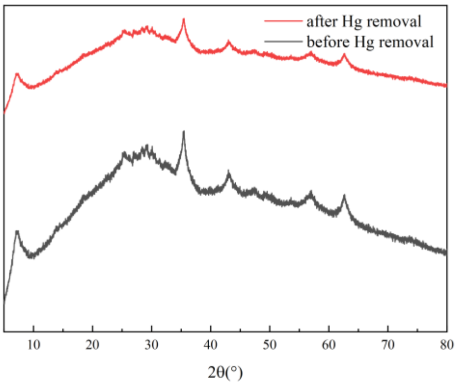


(i) UiO-66-Br@MBC（1:5）

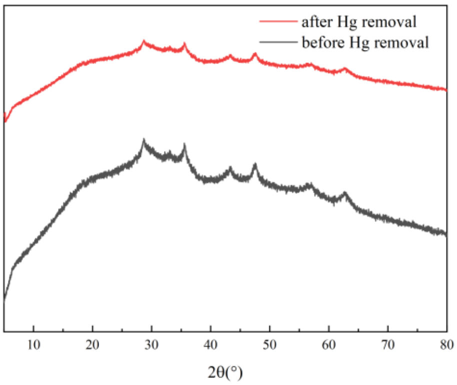


(j) UiO-66-Br@MBC（1:9）

**Figure S6 Comparison of the PXRD patterns of the samples before and after Hg removal**
